# Impact of *Spodoptera frugiperda* (Lepidoptera: Noctuidae), on maize yield in humid tropical zones of Central Africa

**DOI:** 10.1093/jee/toae102

**Published:** 2024-05-20

**Authors:** Komi Mawufe Agbodzavu, Samuel Nanga Nanga, Albert Fomumbod Abang, Apollin Fotso-Kuate, Zoumana Bamba, Cargele Masso, Komi Kouma Mokpokpo Fiaboe

**Affiliations:** Plant Health Department, IITA-Democratic Republic of Congo, C/Gombe, 4163 Avenue, Du Haut Congo, Kinshasa, Democratic Republic of Congo; Plant Health Department, IITA-Cameroon, P.O. Box. 2008 (Messa), IRAD Main Road, Nkolbisson, Yaoundé, Cameroon; Plant Health Department, IITA-Cameroon, P.O. Box. 2008 (Messa), IRAD Main Road, Nkolbisson, Yaoundé, Cameroon; Plant Health Department, IITA-Cameroon, P.O. Box. 2008 (Messa), IRAD Main Road, Nkolbisson, Yaoundé, Cameroon; Plant Health Department, IITA-Democratic Republic of Congo, C/Gombe, 4163 Avenue, Du Haut Congo, Kinshasa, Democratic Republic of Congo; Plant Health Department, IITA-Cameroon, P.O. Box. 2008 (Messa), IRAD Main Road, Nkolbisson, Yaoundé, Cameroon; Plant Health Department, IITA-Cameroon, P.O. Box. 2008 (Messa), IRAD Main Road, Nkolbisson, Yaoundé, Cameroon

**Keywords:** agroecology, damage incidence, damage severity, yield, cost–benefit

## Abstract

Fall armyworm, *Spodoptera frugiperda* Smith, became the most important maize pest in Africa in 2016, with management based on chemical pesticides. High yield losses across the continent were predicted based on farmers’ perceptions, but existing agroecological differences were not considered. In the Democratic Republic of Congo, experiments were conducted to assess fall armyworm damage and yield losses in maize farms with and without treatment. The study included 2 seasons in the Kipopo wetland in 2020 and 2021, one rainy season in Kanyameshi in 2021, 2 rainy seasons in Mulungu in 2020 and 2021, and one season in a wetland on the Bishibiru site in 2020. In addition, the research was also conducted at 4 sites in Cameroon from September to December 2020 and from March to July 2021. High levels of damage incidences were recorded, but the density of larvae per plant was low, with low to moderate levels of damage severities in different seasons and sites. Treatment significantly reduced the number of fall armyworm larvae and their damage severity. However, the high infestation levels did not significantly reduce yield. Cost–benefit ratios were either negative or, in most cases, less than 1. In the best case, the use of pesticides only allowed the recovery of the amount used for the intervention. We discussed the implications of these findings for fall armyworm management in humid tropical agroecology.

## Introduction

Maize (*Zea mays* L.) is a staple food crop in many African countries, accounting for 40% of cereal production in sub-Saharan Africa. Despite its importance, maize yields in farmers’ fields remain significantly low. In several developing countries, particularly in sub-Saharan Africa, the average maize yield is less than 1 tonne per hectare, mainly due to poor soil fertility, frequent droughts, diseases, weeds, and pests. Limited access to fertilizer and farmers’ lack of access to improved maize seeds also contribute to this problem (World Bank 2009, Shiferaw et al. 2011, FAOSTAT 2016, Ekpa et al. 2018, Cairns et al. 2021, Grote et al. 2021, Anjorin et al. 2022). Among the insect pests limiting maize production, lepidopteran stemborers cause huge yield losses in maize. (De Groote 2002, Magdy et al. 2016). For decades, the spotted stemborer *Chilo partellus* (Swinhoe) (Lepidoptera: Crambidae) and the African stemborer *Busseola fusca* (Fuller) (Lepidoptera: Noctuidae) were the 2 most economically important maize pests in Africa (Tefera et al. 2016). However, the invasion of the fall armyworm, *Spodoptera frugiperda* (J. E. Smith) (Lepidoptera: Noctuidae) on the continent in 2016 (Goergen et al. 2016) exacerbated the situation and changed the pest landscape for maize, with the invasive pest widely dominating (Hailu et al. 2021, Sokame et al. 2021). Faced with this situation, many African governments resorted to emergency interventions consisting of the procurement and distribution of nonvalidated insecticides to reduce yield losses (Ahissou et al. 2021, Otim et al. 2021). These interventions are usually guided by a pest incidence-based action threshold (Prasanna 2018, FAO and PPD 2020), but so far, there is no evidence that this is a good decision-making tool. Analyses of expert opinions, farmers’ perceptions, and national statistical data predicted high yield losses across the continent (Day et al. 2017, De Groote et al. 2020) but without taking into account the agroecological differences that exist across the continent. There are also potential errors in yield loss estimates despite known problems with data derived from farmer perceptions or expert opinions. In addition, the current pesticide-based response to fall armyworm infestation is largely derived from previous experience in the Americas (particularly northeastern Brazil and Florida State in the United States), where the maize cropping system is predominantly commercial, whereas in Africa maize production is driven by small-scale subsistence farmers with limited resources (Niassy et al. 2021). It is important to note that the lessons learned from agroecological conditions in northeastern Brazil and the US state of Florida are not necessarily applicable to the African situation. This is because most of the maize production in Brazil (86.3%) is concentrated in 4 states dominated by savannah and dry forest agroecologies (MAPA 2019). Therefore, after the panic and emergency phase that followed the invasion of *S*. *frugiperda* in Africa and Asia, there was a need to conduct regional and local studies to understand the pest-specific dynamics, the impact on maize productivity and production and to validate the efficacy of technologies used in the Americas.

Several attempts have been made to assess yield and national production losses in African countries since the invasion of *S. frugiperda*. However, these assessments were mainly based on community surveys and questionnaires (Day et al. 2017, Kansiime et al. 2019, Kumela et al. 2019, De Groote et al. 2020) but rarely on direct biophysical data collection (Baudron et al. 2019). For a pest like *S. frugiperda*, whose main damage is defoliation of maize with comparatively less damage to other parts of the plant, unlike maize stem and ear borers, especially the African stalk borer, which can cause equal damage to leaves, stems, and ears, overestimation of damage and in particular yield impacts is likely to occur when yield losses are estimated based on farmers’ observations (Overton et al. 2021). While leaf damage by *S. frugiperda* can be alarming, it is important to note that maize can recover from severe leaf damage depending on agroecological conditions and farming practices (Tshiabukole et al. 2021). Therefore, it is understandable that Baudron et al. (2019) estimated yield losses as low as 11.57% under savannah conditions in Zimbabwe using direct measurements. Biophysical yield loss assessments are time-consuming and costly but are necessary for informed pest management policy formulation in diverse agroecological conditions.

While there is no data available on the relationship between foliar damage and yield loss under African growing conditions, most literature from North and South America indicates little to no correlation between the level of *S*. *frugiperda* infestation, foliar damage, and actual yield loss (van den Berg et al. 2021). This suggests that factors other than foliar damage play a critical role in actual yield losses. Studies have shown that maize can recover or tolerate fall armyworm infestations when fertilizer is applied (Kansiime et al. 2019). Crops that are not stressed by drought can resist or recover from fall armyworm attacks much better (FAO 2018a, Hruska 2019). Central Africa benefits from a large (2.69 million km^2^) area of the Congo Basin Forest, which provides a tropical humid forest agroecology (CBFP 2005) characterized by high annual rainfall, ranging on average from 1,000 m to 4,500 mm/year, with areas such as Debundscha in southwest Cameroon known as the second highest rainfall zone in the world with up to 11,000 mm/year (Wasseige et al. 2015, ONACC 2028). The effect of rainfall *on S. frugiperda* has been demonstrated by Sims (2008), where increasing simulated rainfall from 0 to 8 cm significantly reduced adult emergence of the fall armyworm; heavy rainfall reduces adult emergence by trapping moths in their pupation tunnel, and more fragile soils, rainfall caused the soil to collapse. The larval population has also been shown to be affected by the amount of rainfall (Silvain and Ti-A-Hing 1985). Nboyine et al. (2020) hypothesized that rainfall first leads to the flourishing growth of host plants, thereby creating the conditions for the growth of the fall armyworm population, and at the same time, rainfall and irrigation wash away or drown the fall armyworm. The aim of this study was, therefore, to conduct biophysical data-based experiments in the humid tropical agroecological zones of Cameroon and the Democratic Republic of Congo to assess yield losses in untreated maize compared to widely used and effective active ingredients against *S. frugiperda*. This study will guide agroecosystem-specific development of environmentally sustainable management of *S. frugiperda* in the target countries and, by extrapolation, across the humid tropical agroecology of Africa and beyond.

## Materials and Methods

### Study Site

*Cameroon*. The field experiment took place in 2 districts in the same bimodal rainfall humid tropical forest agroecological zone but with different agro-ecosystems. These districts were Mbalmayo and Batchenga, 87 km apart. Mbalmayo, located in a forest zone in the south of Cameroon, has a warm and humid climate. The experimental sites were surrounded by forest and cocoa farms, with very few maize farms nearby. Two different sites were used, consisting of rented farmers’ fields 16 km apart. Both sites had the same conditions before the experiment. They were the villages of Avebe (N 03.36471°, E 011.50853°, 673 m a.s.l.) and Memiam (N 03.4701°, E 011.58514°, 657m a.s.l.) villages, both with a fallow period of 3–4 years and on dryland. During the 12 wk of the experiment in 2020, 867 mm of rain and an average daily temperature of 23.5 °C were recorded, while in 2021, a total of 629 mm of rain and an average daily temperature of 24.1 °C were recorded. The 2 rainy seasons in this district occur from mid-March to mid-June for the small rainy season and from mid-August to mid-November for the heavy rainy season, while the long dry season occurs from mid-November to mid-March and the short dry season from mid-June to mid-August, with annual rainfall of 1,918 and 1,718 mm in 2020 and 2021, respectively.

The second district, Batchenga, lies to the north, is considered a transitional zone between forest and savannah, and has relatively low rainfall. This district is a major producer of maize, and the experimental sites were all located in the middle of large areas of small-scale maize farms. Two different sites, Okongolo village (N 04.25423°, E 11.65100°, 510 m a.s.l.) and Ndjie village (N 04.34797°, E 11.68201°, 490 m a.s.l.), were selected with a fallow period of 1-2 years. They were rented farmer’s fields 9 km apart, the first site being close to a small river and the second site closer to a large river. During the 12-wk experiment in 2020, a total of 426 mm of rain was observed, along with an average daily temperature of 25.4 °C. In 2021, a total of 526 mm of rain was recorded, with a temperature of 25.6 °C. The district has 2 seasons: rainy and dry. In 2020 and 2021, the annual rainfall was 1,169 mm and 1,548 mm, respectively. The vegetation type at the Batchenga sites is mainly savannah, with some forest galleries in the vicinity. Maize has 2 main cropping seasons in both districts: the first starts in March to June and the second in September to December. Some farmers in both districts also grow off-season maize in swampy areas with wet soils. For the experiments, 4 sites were established per season, resulting in a total of 8 site seasons.

*Democratic Republic of Congo.* The trials were carried out in 2 provinces separated by 827 km: Haut Katanga and Sud Kivu. The province of Haut Katanga has a long dry and cold season of 6–7 months (April to November), with temperatures ranging from −3 °C to +18 °C (April to August). The rainy season also coincides with the main maize growing season, from November to March, although some farmers grow maize outside the season. The average annual temperature is around 19 °C, while the average annual rainfall varies between 900 and 1,400 mm/year. December is the wettest month, and the province has a semi-humid agroecology. In Sud Kivu, the dry season lasts 3 to 4 months, from June to September. The rainy season lasts 9 months, from September/October to May, with an average annual temperature of 19 °C and annual rainfall of 1,400 mm. It is common to experience a dry spell in February, while rarely any rainfall occurs during the dry season in July is rare. Maize is grown on smallholders’ fields from September to February. From March to May, grain legumes replace maize production, although a few farmers may still grow maize during this period for home consumption and/or sale as green maize. In Haut Katanga, the trials were established at the national research stations at Kipopo (S 11° 33.941ʹ; E 027° 21.756ʹ; 1,258 m a.s.l.), wetland, and Kanyameshi (S 11° 45. 553ʹ; E 027° 16.332ʹ; 1,315 m a.s.l.) dryland, with a distance of 23.7 km apart, while in Sud Kivu they were established in the national research station at Mulungu (S 02° 20ʹ 118″; E 028° 47ʹ 483″; 1679 m a.s.l.) dryland, and a rented farmer field with wetland in Bishibiru village (S 2,257297°; E 28,811470°; 1673,85 m a.s.l.) with a distance of 8.04 km apart. The agroecology in Sud Kivu is a humid tropical forest at high altitudes. In Haut Katanga, 2 sites were used, one of which had 2 seasons, making a total of 3. Similarly, in Sud Kivu, one of the 2 sites was used in 2 different seasons, giving a total of 3 sites and, therefore, 6 site-seasonal data for the country.

### Experimental Design and Field Establishment

In Cameroon, the trials were conducted in the 2 districts during the second rainy season, from September to December 2020, and during the first rainy season of 2021, from March to June. For the 2020 first season, planting took place on 8 and 9 September at the Njie and Okongolo sites (Batchenga District) and on 14 and 15 September at the Memiam and Avebe sites (Mbalmayo District), while for the 2021 season, planting took place on 18 and 19 March at the Njie and Okongolo sites and on 23 and 24 March 2021 at the Memiam and Avebe sites, after it had been confirmed that the first rains had provided good conditions for the start of the trial. In the Democratic Republic of Congo, in Haut Katanga, validation trials were established in Kipopo (wetland) during a mixed dry and rainy season, planted on 29 August 2020, and during the dry cold season, planted on 17 March 2021. In Kanyameshi, the rainy season trials were planted on 9 December 2020. In Sud Kivu, the trials were established in Mulungu (dryland) and Bishibiru (wetland) on 7 and 21 September 2020, respectively, and in Mulungu (dryland) during the short rainy season on 18 March 2021.

The objectives of the trial were to assess the yield losses caused by *S. frugiperda* and to compare the efficacy of an insecticide against the pest under field conditions. The insecticide emamectin benzoate is registered against *S. frugiperda* in Cameroon as GREMEC 050 WG and in the Democratic Republic of Congo as EMACOT 050 WG. The untreated plots consisted of normal maize production plots with no insecticide application. The trial was designed as a randomized complete bloc design with 4 replications at each experimental site. Each 10 m × 10 m plot was separated by 2 m and blocks by 5 m. Fields were manually cleared, debris was removed, and the land was plowed before planting. Manual weeding was carried out each season.

In Cameroon, the fields were planted with a 3-month cycle CMS 8704 maize seed, the most commonly used variety by farmers in both districts. Row and inter-row spacing was 50 cm × 50 cm, which is in line with national recommendations for maize production in the area. In the Democratic Republic of Congo, maize varieties MUDISHI 1 (used for all 6 sites—seasons) and MUDISHI 3 (used for 2 sites—seasons, the second seasons in Mulungu and Kipopo), open-pollinated varieties with 5 months and 3 months maturity respectively, were used and planted at a density of 45 cm within rows and 75 cm between rows with 3 seeds per hole, following national recommendations for the area. Two weeks after planting (WAP), the crop was thinned to one plant per stand in Cameroon and 2 plants per stand in the Democratic Republic of Congo.

In Cameroon, a mixture of NPK (20-10-10) and urea (3:1) was applied to maize 2 WAP, alongside thinning, at approximately 11g per plant. Two weeks later, the second split dose of urea was applied at the same rate. For weed control, a pre-emergence herbicide, glyphosate (Quiclear Super, 620g/L), was applied 1 day before planting, followed by hand weeding as necessary.

In the Democratic Republic of Congo, basal fertilization consisted of diammonium phosphate (DAP) at planting at a rate of 150 kg/ha, while top dressing fertilization was applied 45 days after planting (DAP) with urea at a rate of 100 kg/ha in Sud Kivu and 300 kg/ha in Haut Katanga, following recommendations for each province. Manual weeding was carried out as necessary.

### Treatment Applications

As planned for each site, interventions were only implemented when at least 10% (pesticide intervention threshold) of fall armyworm damage incidence (percentage of damaged plants out of the total number of plants) was reported in at least 1 plot (Prasanna et al. 2018). In Cameroon, this threshold was reached at 4 WAP. In the Democratic Republic of Congo, this threshold was also reached at 4 WAP for Kipopo 1st season, Mulungu 1st season, and Bishibiru 1st season, but 2 WAP for Kanyameshi 1st season and 3 WAP for Kipopo 2nd season and Mulungu 2nd season.

In Cameroon, the synthetic insecticide was applied twice at the recommended rate of 300 g/ha at 10 g/sprayer in 15 L of water, first at 4 WAP and then at 6 WAP. The number of sprayers was doubled at 6 WAP due to an increase in plant size. In the Democratic Republic of Congo, the recommended rate of 250 g/ha was applied twice at 10 g/sprayer of 15 L, first when thresholds were reached at each site. At all sites, the second treatment was applied 2 wk after the first. Similarly, in the Democratic Republic of Congo, both treatments were applied during the vegetative stage, when the plants had not started to tassel. Treatment was applied as a full-cover spray at all sites.

### Sampling and Data Collection

Data were collected every 2 wk. The damage incidence, the damage severity, and the larval abundance were recorded. The damage incidence and the damage severity were recorded on 22 and 20 plants randomly selected along the 2 diagonals in Cameroon and the Democratic Republic of Congo, respectively. These plants were marked and monitored until harvest. The incidence was recorded as the presence (1) or absence (0) of fall armyworm damage/injury symptoms. It was calculated as the number of plants showing fall armyworm symptoms divided by the total number of plants sampled and multiplied by 100. The damage severity (leaf loss) was scored on a scale of 0–5 (0 = 0% damage; 1 = 1%–20% plant damage; 2 = 21%–40% plant damage; 3 = 41%–60% plant damage; 4 = 61%–80% plant damage; and 5 = 81% to 100% plant damage), a modified method of Carvalho (1970) and adapted from Ghidiu and Drake (1989), by visual inspection of all sampled plants. During the biweekly data collection, destructive sampling was done on 8 other plants selected from the 4 triangles defined by the diagonals, and the number of larvae collected, expressed as larval abundance was calculated as the destructive sampling larval density per plant. The sampling covered the vegetative to reproductive stages.

At maturity, 3 plots or sub-blocks of 2.05 m^2^ and 2.25 m^2^ each were harvested per replicate on a diagonal in the Democratic Republic of Congo and Cameroon, respectively. This corresponded to 6 plant stands, or a maximum of 12 plants, in the Democratic Republic of Congo and 9 plant stands, or 9 plants in Cameroon. Yields were calculated through an adaptation of the method of Blumenthal and Thompson (2009). For each cob harvested from the sample area, the number of rows of grains was counted. The number of grains in the longest and shortest rows was also recorded, and the average number of grains per row was calculated. The average number of grains per row was multiplied by the number of rows to give the number of grains per ear. To determine the number of grains per sampled area, the number of grains for each cob was summed. To calculate the number of grains per hectare, the number of grains for each sampled area was multiplied by 10,000 m^2^ and divided by 2.05 m^2^ in the Democratic Republic of Congo and by 2.25 m^2^ in Cameroon. To obtain the number of bushels per hectare, the number of grains per hectare was divided by the bushel factor. According to the authors, drought-stressed maize has about 110,000 small grains per bushel, compared with 90,000 medium grains per bushel for normal maize and 70,000 large grains per bushel for exceptional maize. Actual kernel sizes were determined by measuring a sample of kernels. The category of the grains was determined using a key to determine the “grains per bushel factor graph.” To obtain the yield in tonnes per hectare, the number of bushels per hectare was multiplied by 25.4/1,000. Note: 1 bushel is equal to 25.4 kg.

### Cost and Benefit of Insecticide Application

The costs of the different technologies were estimated per hectare. This estimate took into account the cost of the insecticides, the labor to apply the insecticides, the depreciation of the sprayer, the PPE, the containers, and the water used. In the Democratic Republic of Congo, a 10 g sachet of emamectin benzoate costs $0.04. Based on the recommended rate per hectare, the cost of treating 1 ha was $10.00, and for 2 applications per season, the cost was $20.00. The cost of the application in Sud Kivu was $16.00 per ha, i.e., $32.00 for the 2 applications, while in Haut Katanga, it was $28.60 per ha, i.e., $57.20 for the 2 applications. The depreciation of the sprayer was calculated at $0.07/ha for Sud Kivu and $0.03 for Haut Katanga, i.e., $0.13 and $0.07 for the 2 applications in Sud Kivu and Haut Katanga, respectively. According to Phillippe et al. (1983), a sprayer can treat 150 ha per year and can be used for 3 years. We used the same approach for the container and personal protective equipment (PPE), except that they can only be used for 1 year. The depreciation of the containers used was then $0.33 for the 2 applications ($0.17/ha). The depreciation of PPE was $0.45 ($0.22/ha) and $0.37 ($0.19/ha) for Sud Kivu and Haut Katanga, respectively. Water costs were $12.50 ($6.25/ha) and $15.00 ($7.50/ha) in Sud Kivu and Haut Katanga, respectively. The total cost of 2 sprays in Sud Kivu and Haut Katanga was $65.41 and $92.97 respectively.

In Cameroon, the cost of emamectin benzoate was $0.84 per 10 g sachet, giving $25.20 per ha and $50.40 for the 2 applications. The cost of spraying the suspension was $25.20 per hectare, giving a cost of $50.40 for the 2 applications. The depreciation of the containers used was $0.33 for the 2 applications ($0.17/ha). The depreciation of the sprayer was calculated at $0.18/ha, which was $0.36 for the 2 applications. The PPE depreciation was $0.75 ($0.37/ha). The cost of water was $11.25 ($5.63/ha). The total cost of the 2 applications was $113.32.

To determine whether pesticide application increases or decreases profits, a partial budget analysis approach was used. This approach assesses the costs and benefits associated with an action or a specific change in a single enterprise within the business operation (Soha 2014). Partial budget analysis does not include all production costs but only those that change or vary between the farmer’s current production practices and the proposed practice(s). It allows us to assess the impact of a change in the production system on a farmer’s net income without knowing all his production costs. To carry out this analysis, the farm gate income was determined by multiplying the production/yield under each treatment by the unit price of maize grain. A mean value of $0.225/kg and $0.361/kg were used for the unit prices for the Democratic Republic of Congo and Cameroon, respectively, based on farm gate prices in the experimental plots in 2021. The cost of spraying was subtracted from the farm gate income for adopting chemical treatment to arrive at a projected net return or net income. The net return of spraying was then compared with the untreated net return. It is important to note that a partial budget analysis does not determine profitability; it only determines the “change in profit” if an alternative is adopted. In addition, the cost–benefit ratio was calculated using the following equation (Ponnusamy 2003).


$$\begin{array}{l} Cost\text{-}benef\;\;itratio=\frac{Treatedbenef\;\;it\left(\$\right)-untreatedbenef\;\;it\left(\$\right)}{Costofprotection\left(\$\right)} \end{array}$$

### Data Analysis

To assign a percentage to different fall armyworm plant damage severity levels before analysis, the mid-point of each scale was used. As such, a plant with damage scales of 0 (0% damage on the plant), 1 (1%–20%), 2 (21%–40%), 3 (41%–60%), 4 (61%–80%), and 5 (81%–100%) was assigned a score of 0%, 10.5%, 30.5%, 50.5%, 70.5%, and 90.5% damage severity, respectively. To evaluate the effect of the insecticides, the collected data on fall armyworm larval abundance, damage incidence, and damage severity were analyzed. All data were tested for normality and homogeneity of variance using the Shapiro–Wilk and Bartlett tests, respectively.

To compare damage incidence between treated and untreated plots, a generalized linear mixed effect model (GLMM) with binomial error distribution (link = logit) was fitted to the incidence data using the *glmer* function in the *lme4* package (Bates et al. 2023). The dependent or response variable was damage incidence, while the independent variables were treatment, site, season, and week after planting (WAP). The fixed effects in this model were treatment and site, while the random effects were season and WAP. To compare larval abundance between treated and untreated plots, a GLMM with Poisson error distribution and log link function was fitted to the larval abundance data using the *glmer* function in the lme4 package. The dependent variable was larval abundance, while the independent variables were as indicated above. The same model parameters (fixed and random effects) were used as described above. To compare leaf damage severity between treated and untreated plots, a Generalized Additive Model for Location Scale and Shape (Rigby and Stasinopoulos 2005) with a zero-adjusted gamma distribution was fitted to the damage severity data using the *gamlss* function in the *gamlss* package. This model was used because of the presence of zeros in the damage severity data.

The Anova function from the car package (Fox and Weisberg 2019) was used to test the significance of the fixed effects. The joint tests function in Emmeans (Lenth 2023), which obtains and tests the interaction contrasts for all effects in the models, was used for mean separation.

In addition to these global analyses, site-specific analyses were conducted by WAP. In this case, a generalized linear model (GLM) with binomial and Poisson error distributions and log link functions were fitted to the data to compare damage incidence and larval abundance between treated and untreated plots. In the case of over-dispersion of data, a GLM with a quasi-binomial or quasi-Poisson error distribution was applied. The effect of treatments on leaf damage severity, yield, farm gate income, and net return was analyzed using the Student’s *t*-test, Welch’s *t*-test, or Wilcoxon test. The response variables were damage incidence, larval abundance, damage severity, yield, farm gate revenue, and net return, respectively, and the independent variable was treatment. The significance level was set at 0.05.

All statistical analyses were performed using R version 4.3.1 (R Core Team 2023).

## Results

### Damage Incidence

*Global analyses*. In Cameroon, the fall armyworm damage incidence was significantly affected by the treatment (LR *χ*^2^ = 405.92; *df* = 1; *P* < 0.001), the location (LR *χ*^2^ = 368.28; *df* = 3; *P* < 0.001), and the interaction between the treatment and the location (LR *χ*^2^ = 71.44; *df* = 3; *P* < 0.001). Damage incidences on unsprayed and sprayed plots were higher on Mamian and Avebe (Mbalmayo) (15.03 ± 1.17–59.09% ± 1.62%) compared to Okongolo and Ndjie (Batchenga) (11.83 ± 1.08–25.78% ± 1.46%). Among all the locations, the untreated plots in Avebe registered the highest damage incidence. Generally, damage incidences were significantly higher (*P* < 0.01) on the unsprayed plots compared to the treated plots (Table 1).

**Table 1.** Fall armyworm damage incidence comparison between emamectin benzoate-treated and untreated maize in 4 experimental sites in the humid forest of Cameroon over 2 cropping seasons

Agroecology	Location/field	Treatments	Damage incidence (%)	*Z*, *P*
Forest (Mbalmayo)	Avebe	Untreated	59.09 ± 1.62 b	16.31; <0.0001
		Treated	22.39 ± 1.37 a	
	Memiam	Untreated	41.75 ± 1.61 b	12.90; <0.0001
		Treated	15.03 ± 1.17 a	
Mean Mbalmayo			34.60 ± 0.78	
Transitional zone (Batchenga)	Ndjie	Untreated	25.78 ± 1.46 b	6.30; <0.0001
		Treated	14.17 ± 1.165 a	
	Okongolo	Untreated	17.67 ± 1.27 b	3.56; 0.0004
		Treated	11.83 ± 1.08 a	
Mean Batchenga			17.36 ± 0.63	

Means comparison between the treated and untreated plots was done within each location.

In the Democratic Republic of Congo, damage incidence was also significantly affected by the treatment (LR *χ*^2^ = 127.70; *df* = 1; *P* < 0.001), the location (LR *χ*^2^ = 83.37; *df* = 3; *P* < 0.001), as well as the interaction between the treatment and the location (LR *χ*^2^ = 73.32; *df* = 3; *P* < 0.001). Damage incidences are generally very high in all locations, with the highest on untreated plots in Mulungu (Sud Kivu; 98.47% ± 0.46%); the effect of the treatments was dependent on the location (Table 2).

**Table 2.** Fall armyworm damage incidence comparison between Emamectin benzoate-treated and untreated maize in 4 experimental sites in the humid forest/humid savannah of DR Congo over 2 cropping seasons

Province	Location	Treatments	Damage incidence (%)	*Z*, *P*
Haut Katanga	Kipopo	Untreated	83.75 ± 1.3 a	−1.64; 0,101
		Treated	61.12 ± 1.72 a	
	Kanyameshi	Untreated	85.83 ± 1.59 b	7.24;<0.001
		Treated	66.04 ± 2.16 a	
Mean Haut Katanga			73.75 ± 0.87	
Sud Kivu	Bishibiru	Untreated	81.5 ± 1.94 b	11.71;<0.001
		Treated	85.75 ± 1.75 a	
	Mulungu	Untreated	98.47 ± 0.46 b	3.055; 0.002
		Treated	95.69 ± 0.76 a	
Mean Sud Kivu			92.27 ± 0.56	

Means comparison between the treated and untreated plots was done within each location.

*Site-specific analyses*. The fall armyworm damage incidence in the untreated was very highly significant (LR *χ*^2^ = 17.17; *df* = 1; *P* < 0.001—LR *χ*^2^ = 7.68; *df* = 1; *P* = 0.005), significant (LR *χ*^2^ = 6.53; *df* = 1; *P* = 0.011—LR *χ*^2^ = 4.04; *df* = 1; *P* = 0.044), or similar (LR *χ*^2^ = 2.42; *df* = 1; *P* = 0.119—LR *χ*^2^ = 0.277; *df* = 1; *P* = 0.599) to treated plots in 50.00%, 12.50%, or 37.50% of the cases, respectively when the data where considered per sites and the sampling dates. This represents a total success rate in damage incidence, with a significant reduction of 62.50%. At Mbalmayo, where higher damage incidences were recorded, in 81.25% of the cases, the incidence was very significantly higher (LR χ^2^ = 17.17; *df* = 1; *P* < 0.001—LR *χ*^2^ = 7.68; *df* = 1; *P* = 0.005) in the untreated compared to the treated plots, while at Batchenga where lower incidences were recorded, no significant difference was found between the untreated and treated plots in 56.25% of cases (LR *χ*^2^ = 1.7; *df* = 1; *P* = 0.192—LR *χ*^2^ = 0; *df* = 1; *P* = 1) with only 18.75% and 25.00% of the cases with highly significant (LR *χ*^2^ = 35.21; *df* = 1; *P* < 0.001) and significant differences (LR *χ*^2^ = 6.53; *df* = 1; *P* = 0.011—LR *χ*^2^ = 4.04; *df* = 1; *P* = 0.044) in damage incidences respectively in the untreated compared to treated plots (Table 3).

**Table 3.** Fall armyworm damage incidence comparison between Emamectin benzoate-treated and untreated maize in 4 experimental sites at different sampling dates in the humid forest of Cameroon over 2 cropping seasons

WAP	Treatment	Mbalmayo district				Batchenga district			
		Memiam 2020	Memiam 2021	Avebe 2020	Avebe 2021	Ndjie 2020	Ndjie 2021	Okongolo 2020	Okongolo 2021
4	Untreated	55.08 ± 4.6a	39.28 ± 4.63a	31.82 ± 4.46	50.00 ± 4.74	1.78 ± 1.26	40.18 ± 4.65	14.55 ± 3.38	18.75 ± 3.7
	Treated	37.07 ± 4.5b	30.36 ± 4.36b	42.06 ± 4.79	39.28 ± 4.63	1.78 ± 1.26	39.28 ± 4.63	19.64 ± 3.77	13.39 ± 3.23
	LR χ2*; df; P*	*7.62; 1; 0.006*	*7.68; 1; 0.005*	*2.42; 1; 0.119*	*2.42; 1; 0.119*	*0; 1; 1*	*0; 1; 1*	*1.01; 1; 0.315*	*1.01; 1; 0.315*
6	Untreated	33.9 ± 4.38a	61.67 ± 4.46a	60.34 ± 4.56a	89.17 ± 2.85a	18.75 ± 3.70	51.78 ± 0.05a	18.75 ± 3.7	38.39 ± 4.62
	Treated	11.67 ± 2.94b	16.95 ± 3.49b	15.97 ± 3.37b	43.22 ± 4.58b	16.07 ± 3.48	35.71 ± 0.05b	16.07 ± 3.48	32.14 ± 4.43
	LR χ2*; df; P*	*17.17; 1; < 0.001*	*51.757; 1; < 0.001*	*51.06; 1; < 0.001*	*59.66; 1; < 0.001*	*0.277; 1; 0.599*	*5.85; 1; 0.016*	*0.28; 1; 0.599*	*0.95; 1; 0.330*
8	Untreated	31.3 ± 4.34a	60.00 ± 4.49a	41.59 ± 4.66a	90.83 ± 2.64a	8.03 ± 2.58a	45.53 ± 4.73a	6.25 ± 2.3	25.89 ± 4.16a
	Treated	3.54 ± 1.75b	15.79 ± 3.43b	3.42 ± 1.69b	21.19 ± 3.78b	1.78 ± 1.26b	10.71 ± 2.94b	4.46 ± 1.96	12.5 ± 3.14b
	LR χ2*; df; P*	*33.93; 1; < 0.001*	*50.42; 1; < 0.001*	*54.6; 1; < 0.001*	*129.64; 1; < 0.001*	*5; 1; 0.025*	*35.21; 1; < 0.001*	*0.35; 1; 0.554*	*6.53; 1; 0.011*
11	Untreated	14.65 ± 3.30a	36.67 ± 4.42a	19.47 ± 3.74	83.33 ± 3.42a	6.25 ± 2.30	33.93 ± 4.49a	5.36 ± 0.02a	13.39 ± 3.23a
	Treated	2.54 ± 1.45b	2.63 ± 1.51b	11.71 ± 3.06	4.24 ± 1.86b	2.68 ± 1.53	5.36 ± 2.14b	0.89 ± 0.01b	0.89 ± 0.89b
	LR χ2*; df; P*	*11.89; 1; < 0.001*	*48.86; 1; < 0.001*	*2.56; 1; 0.120*	*175;59; 1; < 0.001*	*1.7; 1; 0.192*	*31.38; 1; < 0.001*	*4.04; 1; 0.044*	*15.5; 1; < 0.001*

A comparison is made within a year, using the same sampling and between the untreated and the treated plots; values followed by the same letter are not statistically different from the generalized linear model with a binomial error distribution (*P* = 0.05). WAP, weeks after planting.

The lowest damage incidence of 0.89 ± 0.01 was recorded in the treated plots at the Okongolo site in the Batchenga district both in 2020 and 2021 at the most advanced growth stage of 11 WAP (week after planting). The highest damage incidence was 90.83 ± 2.64 in the untreated at 8 WAP at the Avebe site in the Mbalmayo district in 2021. At Mbalmayo, the untreated recorded less than 20% damage incidence only in 12.50% of cases, while the treated plots recorded it in 62.50% of the cases. At Mbalmayo, where lower incidences were recorded, the untreated and the treated recorded less than 20% incidence in 62.50 and 81.25% of cases, respectively (Table 3).

At Mbalmayo, when combining both years for each site, Avebe site recorded 59.09 ± 1.6 and 22.39% ± 1.37% damage incidence for the untreated and treated plots, respectively (LR*χ*^2^ = 264.23, *df* = 1; *P* < 0.001), while Memiam site recorded 41.75 ± 1.61 and 15.02 ± 1.17% incidence, respectively (LR*χ*^2^ = 168.38; *df* = 1; *P* < 0.001). At Batchenga, in the same conditions, the Ndjie site recorded 25.78 ± 1.46 and 14.17% ± 1.16% incidence for the untreated and treated plots, respectively (LR*χ*^2^ = 38.20, *df* = 1; *P* < 0.001), while Okongolo recorded 17.67 ± 12.7 and 11.83% ± 1.07% incidence, respectively (LR*χ*^2^ = 12.22, *df* = 1; *P* < 0.001).

In the Democratic Republic of Congo, damage incidence in the untreated was very highly significant (LR*χ*^2^ = 12.28; *df* = 1; *P* < 0.001—LR*χ*^2^ = 8.19; *df* = 1; *P* = 0.004), significantly higher (LR*χ*^*2*^ = 5.47; *df* = 1; *P* = 0.019—LR*χ*^2^ = 4.23; *df* = 1; *P* = 0.039), or similar (LR*χ*^2^ = 2.8; *df* = 1; 0.094—LR*χ*^2^ = 0; *df* = 1; *P* = 1) to treated plots in 50.00%, 6.67%, or 43.33% of the cases, respectively. This represents a 56.67% success rate in a significant reduction of fall armyworm damage incidence. In the Haut Katanga province, the untreated was highly significantly higher (LR*χ*^2^ = 12.28; *df* = 1; *P* < 0.001—LR*χ*^2^ = 9.42; *df* = 1; *P* = 0.002), significantly higher (LR*χ*^2^ = 5.47; *df* = 1; *P* = 0.019—LR*χ*^2^ = 4.23; *df* = 1; *P* = 0.039), or similar (LR*χ*^2^ = 2.75; *df* = 1; *P* = 0.097—LR*χ*^2^ = 0; *df* = 1; *P* = 1) to treated plots in 62.50%, 6.25%, or 31.25% of the cases, respectively, making 68.75% of the total success rate (Table 4). In Sud Kivu, however, a lower success rate of 42.86% was achieved, with 35.72%, 7.14%, or 57.14% of cases having the untreated highly significant (LR*χ*^2^ = 8.33; *df* = 1; *P* = 0.004), significantly higher (LR*χ*^2^ = 5.52; *df* = 1; *P* = 0.019) or similar (2.8; 1; 0.094–0.15; 1; 0.699) damage incidence levels, respectively, compared to treated plots.

**Table 4.** Fall armyworm damage incidence comparison between Emamectin benzoate-treated and untreated maize in 4 experimental sites at different sampling dates in the humid forest/humid savannah of DR Congo over 2 cropping seasons

Treatment	Haut Katanga Province						Sud Kivu Province					
	Kanyameshi 2020		Kipopo 2020		Kipopo 2021		Bishibiru 2020		Mulungu 2020		Mulungu 2021	
	WAP	Incidence (%)	WAP	Incidence (%)	WAP	Incidence (%)	WAP	Incidence (%)	WAP	Incidence (%)	WAP	Incidence (%)
Untreated	4	58.75 ± 5.54a	6	51.25 ± 5.62a	6	96.25 ± 2.14	6	52.50 ± 5.62a	8	100.00 ± 0.00a	5	88.75 ± 3.55
Treated		36.25 ± 5.41b		13.75 ± 3.87b		92.5 ± 2.96		96.25 ± 2.14b		98.75 ± 1.25b		93.75 ± 2.72
LR χ^2^*; df; P*		*8.19; 1; 0.004*		*26.533; 1; < 0.001*		*1.06; 1; 0.302*		*45.82; 1; < 0.001*		*2.75; 1; 0.10*		*1.25; 1; 0.263*
Untreated	6	92.50 ± 2.96a	8	70.00 ± 5.16a	8	98.75 ± 1.25	8	95.00 ± 2.45	8	100.00 ± 0.00a	6	97.50 ± 1.76
Treated		75.00 ± 4.87b		20.00 ± 4.50b		98.75 ± 1.25		96.25 ± 2.14		96.25 ± 2.14b		98.75 ± 1.25
LR χ^2^*; df; P*		*9.42; 1; 0.002*		*41.872; 1; < 0.001*		*0; 1; 1*		*0.15; 1; 0.699*		*8.33; 1; 0.004*		*0.34; 1; 0.559*
Untreated	8	95.00 ± 2.45a	10	60.00 ± 5.36a	10	98.75 ± 1.25	10	100.00 ± 0.00	10	100.00 ± 0.00a	8	100.00 ± 0.00a
Treated		76.25 ± 4.79b		20.00 ± 4.50b		100 ± 0.00		97.50 ± 1.76		97.50 ± 1.76b		97.5 ± 1.76b
LR χ^2^*; df; P*		*12.28; 1; < 0.001*		*34.11; 1; < 0.001*		*2.75; 1; 0.097*		*2.8; 1; 0.094*		*5.52; 1; 0.190*		*5.52; 1;* 0.019
Untreated	10	91.25 ± 3.18a	12	80.00 ± 4.50a	12	97.5 ± 1.76	12	100.00 ± 0.00	12	100.00 ± 0.00 a	10	100.00 ± 0.00a
Treated		70.00 ± 5.16b		41.25 ± 5.53b		98.75 ± 1.25		97.50 ± 1.76		91.25 ± 3.18 b		96.25 ± 2.14b
LR χ^2^*; df; P*		*12.1; 1; < 0.001*		*25.7; 1; < 0.001*		*0.34; 1; 0.559*		*2.8; 1; 0.094*		*19.8; 1; < 0.001*		*8.327; 1; 0.004*
Untreated	12	86.25 ± 3.87a	14	83.75 ± 4.15a	14	96.25 ± 2.14a	14	60.00 ± 5.51a	14	100.00 ± 0.00a		
Treated		71.25 ± 5.09b		38.75 ± 5.48b		87.5 ± 3.72b		41.25 ± 5.54b		91.25 ± 3.18b		
LR χ^2^*; df; P*		*5.47; 1; 0.019*		*35.36; 1; < 0.001*		*4.23; 1; 0.039*		*5.5886; 1; 0.180*		*19.8; 1; < 0.001*		
Untreated	14	91.25 ± 3.18 a										
Treated		67.50 ± 5.27 b										
LR χ^2^*; df; P*		14.49; 1; < 0.001										

A comparison is done within a year with the same WAP and between untreated and treated plots; values followed by the same letter are not statistically different from the generalized linear model with a binomial error distribution (*P* = 0.05). WAP, weeks after planting.

The untreated and treated fields, respectively, recorded above 50% of damage incidence at 100% and 76.67% of the occasions, showing high damage incidence across all experimental sites with or without intervention (Table 4).

At Haut Katanga, when considering sites, the Kanyameshi site recorded 85.83% ± 3.49 and 66.04% ± 2.16% damage incidence for the untreated and treated plots, respectively (LR*χ*^2^ = 52.56; *df* = 1; *P* < 0.001), while Kipopo recorded 83.75% ± 1.30% and 61.12% ± 1.72% damage incidence, respectively (LR*χ*^2^ = 104.78; *df* = 1; *P* < 0.001). At Sud Kivu, the Bishibiru site recorded 81.50% ± 1.94% and 85.75% ± 1.75% damage incidence for the untreated and treated plots, respectively (LR*χ*^2^ = 2.64, *df* = 1; *P* = 0.104), while the Mulungu site recorded 98.47% ± 0.45% and 95.69% ± 0.75% damage incidence, respectively (LR*χ*^2^ = 10.19, *df* = 1; *P* = 0.0014).

While in Cameroon, at all experimental sites, the threshold of 10% of damage incidence was reached uniformly at 4 WAP; in DRC, the sites at Sud Kivu uniformly reached a 10% threshold at 6 WAP in all the seasons, while in Haut Katanga, a high variation was noted between and within sites, with Kanyameshi reaching the threshold at 4 WAP; and at Kipopo, this was reached at 6 WAP during the rainy season in 2020 but at 2 WAP during the dry season in 2021 (Tables 3 and 4).

### Larval Abundance Based on Destructive Sampling

*Global analyses*. In Cameroon, the treatment (LR*χ*^2^ = 13.15; *df* = 1; *P* < 0.001) and the location (LR*χ*^2^ = 61.96; *df* = 3; *P* < 0.001) had a significant effect on the larval abundance. However, the interaction between the treatment and the location (LR*χ*^2^ = 2.71; *df* = 3; *P* = 0.438) was not significant. The pesticide application significantly reduced the larval abundance (*P* < 0.001). Similarly, Avebe recorded a significantly higher (*P* < 0.001) larval abundance as compared to the other locations. However, the larval abundance was extremely low (<1 larva/plant) (Table 5).

**Table 5.** Fall armyworm larval abundance comparison between Emamectin benzoate-treated and untreated maize in 4 experimental sites in the humid forest of Cameroon over 2 cropping seasons

Factors	Treatments/levels	Larval abundance (larva/plant)
Treatment	Untreated	0.14 ± 0.01 b
	Treated	0.09 ± 0.01 a
Location	Avebe	0.22 ± 0.03 c
	Memiam	0.11 ± 0.02 b
	Mean Mbalmayo	0.16 ± 0.01
	Ndjie	0.09 ± 0.01 ab
	Okongolo	0.05 ± 0.01 a
	Mean Batchenga	0.07 ± 0.01

Means comparisons were made for each factor.

In the Democratic Republic of Congo, the treatment (LR*χ*^2^ = 166.75; *df* = 1; *P* < 0.001), the location (LR*χ*^2^ = 95.50; *df* = 3; *P* < 0.001), and the interaction between the treatment and the location (LR*χ*^2^ = 23.07; *df* = 3; *P* < 0.001) had a significant effect on the larval abundance. As for all the locations in Cameroon, the larval abundance in all locations in the DRC was very low (<1 larva/plant) (Table 6).

**Table 6.** Fall armyworm larval abundance comparison between Emamectin benzoate-treated and untreated maize in 4 experimental sites in the humid forest/humid savannah of DR Congo over 2 cropping seasons

Province	Location	Treatments	Larval abundance (larva/plant)	*Z*, *P*
Haut Katanga	Kipopo	Untreated	0.67 ± 0.08 b	4.63;<0.0001
		Treated	0.2 ± 0.07 a	
	Kanyameshi	Untreated	0.34 ± 0.05 a	1.38; 0.169
		Treated	0.27 ± 0.05 a	
Mean Haut Katanga			0.37 ± 0.06	
Sud Kivu	Bishibiru	Untreated	0.39 ± 0.05 b	6.51;<0.0001
		Treated	0.11 ± 0.03 a	
	Mulungu	Untreated	1.39 ± 0.12 b	11.14;<0.0001
		Treated	0.39 ± 0.04 a	
Mean Sud Kivu			0.64 ± 0.04	

Means comparison between the treated and untreated plots was done within each location.

*Site-specific analysis*. Untreated plots (0.14 ± 0.01 larvae/plant) recorded 1.5 times higher larvae than the treated plots (0.09 ± 0.01 larvae/plant). Mbalmayo (0.16 ± 0.02) had a significantly higher (LR*χ*^2^ = 38.98; *df* = 1; *P* < 0.001) larval abundance than Batchenga (0.06 ± 0.01). The destructive sampling revealed an extremely low fall armyworm larval density per plant, with 96.88% of cases (93.75% in the untreated and 100% in treated plots) recording less than 0.5 larva/ plant and 100% with less than 1 larva/plant (Table 7). With such low levels of larval density, when the sites and the sampling dates are considered, there was no significant difference (LR*χ*^2^ = 2.91; *df* = 1; *P* = 0.090—LR*χ*^2^ = 0; *df* = 1; *P* = 1) between the untreated and treated plots in 75.00% of cases (56.25% in Mbalmayo, where more fall armyworm attacks were recorded, and 93.75% in Batchenga, where fewer attacks were recorded).

**Table 7.** Fall armyworm larval abundance comparison between Emamectin benzoate-treated and untreated maize in 4 experimental sites at different sampling dates in the humid forest of Cameroon over 2 cropping seasons

WAP	Treatments	Mbalmayo district				Batchenga district			
		Memiam 2020	Memiam 2021	Avebe 2020	Avebe 2021	Ndjie 2020	Ndjie 2021	Okongolo 2020	Okongolo 2021
4	Untreated	0.37 ± 0.12	0.37 ± 0.09a	0.34 ± 0.12	0.72 ± 0.23	0.00 ± 0.00	0.28 ± 0.13	0.03 ± 0.03	0.03 ± 0.03
	Treated	0.47 ± 0.17	0.09 ± 0.05b	0.37 ± 0.10	0.22 ± 0.09	0.03 ± 0.03	0.19 ± 0.07	0.03 ± 0.03	0.16 ± 0.10
	LR; χ^2^*; df; P*	*0.33; 1; 0.563*	*7.32; 1; 0.007*	*0.043; 1; 0.835*	*8.99; 1; 0.002*	*2.77; 1; 0.096*	*0.60; 1; 0.437*	*0; 1; 1*	*2.911; 1; 0.088*
6	Untreated	0.06 ± 0.04a	0.19 ± 0.07a	0.44 ± 0.00a	0.59 ± 0.11	0.09 ± 0.07	0.16 ± 0.07	0.09 ± 0.07a	0.19 ± 0.08
	Treated	0.00 ± 0.00b	0.00 ± 0.00b	0.00 ± 0.00b	0.50 ± 0.19	0.03 ± 0.03	0.28 ± 0.09	0.00 ± 0.00b	0.19 ± 0.07
	LR; χ^2^*; df; P*	*5.73; 1; 0.017*	*19.83; 1; < 0.001*	*19.41; 1; < 0.001*	*0.26; 1; 0.612*	*1.04; 1; 0.306*	*1.16; 1; 0.282*	*4.16; 1; 0.041*	*0; 1; 1*
8	Control	0.00 ± 0.00	0.062 ± 0.04	0.03 ± 0.03a	0.03 ± 0.03	0.03 ± 0.03	0.06 ± 0.04	0.00 ± 0.00	0.03 ± 0.03
	Treated	0.00 ± 0.00	0.00 ± 0.00b	0.00 ± 0.00b	0.03 ± 0.03	0.00 ± 0.00	0.00 ± 0.00	0.03 ± 0.03	0.00 ± 0.00
	LR; χ^2^*; df; P*	*0; 1; 1*	*5.73; 1; 0.017*	*1.40; 1; 0.239*	*0; 1; 1*	*1.3863; 1; 0.239*	*1.39; 1; 0.239*	*1.386; 1; 0.239*	*1.386; 1; 0.239*
11	Control	0.09 ± 0.07a	0.00 ± 0.00	0.03 ± 0.03	0.03 ± 0.03	0.19 ± 0.10	0.00 ± 0.00	0.03 ± 0.03	0.00 ± 0.00
	Treated	0.00 ± 0.00b	0.00 ± 0.00	0.17 ± 0.08	0.16 ± 0.08	0.06 ± 0.04	0.00 ± 0.00	0.00 ± 0.00	0.00 ± 0.00
	LR; χ^2^*; df; P*	*4.16; 1; 0.041*	*0; 1; 1*	*2.91; 1; 0.090*	*2.91; 1; 0.09*	*2.093; 1; 0.148*	*0; 1; 1*	*1.386; 1; 0.239*	*0; 1; 1*

A comparison is made within a year and the same sampling, and between the untreated and the treated plots, values followed by the same letter are not statistically different with the generalized linear model with a Poisson or quasi-Poisson error distribution (*P* = 0.05). WAP, weeks after planting.

At Mbalmayo, when combining both years for each site, larval abundance in the Avebe site was significantly higher in the untreated (0.28 ± 0.04 larvae/plant) than in the treated (0.16 ± 0.03 larvae/plant) (LR*χ*^2^ = 8.61; *df* = 1; *P* = 0.003). Similarly, in Memiam, the highest larval abundance was recorded in the untreated (0.13 ± 0.04 larvae/plant) compared to the treated (0.12 ± 0.05 larvae/plant) (LR*χ*^2^ = 5.09; *df* = 1; *P* = 0.024). At Batchenga, in the same conditions, the Ndjie site recorded similar larval abundance in both the untreated (0.10 ± 0.02 larvae/plant) and treated plots (0.07 ± 0.02 larvae/plant) (LR*χ*^2^ = 1.09; *df* = 1; *P* = 0.296). Even in Okongolo, larval abundance was not different between the untreated (0.05 ± 0.25 larvae/plant) and treated (0.05 ± 0.26 larvae/plant) (LR*χ*^2^ = 0.00; *df* = 1; *P* = 1).

In the DRC, the treatment (LR*χ*^2^ = 204.50; *df* = 1; *P* < 0.001), the season (LR*χ*^2^ = 40.71; *df* = 2; *P* < 0.001), the location (LR*χ*^2^ = 70.25; *df* = 1; *P* < 0.001) as well as the interaction between the treatment and season (LR*χ*^2^ = 78.09; *df* = 2; *P* < 0.001), and the interaction between the treatment and location (LR*χ*^2^ = 7.44; *df* = 1; *P* < 0.001) had a significant effect on the larval abundance. Untreated plots (0.76 ± 0.09 larvae/plant) recorded 2.9 times higher larvae than the treated plots (0.26 ± 0.05 larvae/plant). Even though the larval densities were assessed through destructive sampling, their numbers per plant were extremely low, even for destructive sampling of the untreated where no intervention was made, with only 8.00% and 24.00% of cases recording more than 2 and 1 larvae/ plant respectively while 48.00% of cases recorded 0.5 larvae or lower per plant. This is even more pronounced in treated plots where larval densities were constantly below 2 larvae/plant and only 4.00% of cases recorded above 1 larva/plant while 92.00% recorded far below 0.5 larvae/plant. Furthermore, 85.71% of the densities above 1 larva/plant occurred in the single site of Mulungu (Sud Kivu) for both 2020 and 2021 (Table 4). With such low levels in the untreated, in 56.00% of the cases, there was no significant difference (LR*χ*^2^ = 2.99; *df* = 1; *P* = 0.084—LR*χ*^2^ = 0; *df* = 1; *P* = 1) between the untreated and treated plots with regards to larval abundance (Table 8).

**Table 8.** Fall armyworm larval abundance comparison between Emamectin benzoate-treated and untreated maize in 4 experimental sites at different sampling dates in the humid forest/humid savannah of DR Congo over 2 cropping seasons

Treatments	Haut Katanga Province						Sud Kivu Province					
	Kanyameshi 2020		Kipopo 2020		Kipopo 2021		Bishibiru 2020		Mulungu 2020		Mulungu 2021	
	WAP	Abundance	WAP	Abundance	WAP	Abundance	WAP	Abundance	WAP	Abundance	WAP	Abundance
Untreated	4	0.34 ± 0.14		–		–	6	0.50 ± 0.14a	6	0.62 ± 0.10a	6	3.47 ± 0.55a
Treated		0.34 ± 0.14		–		–		0.12 ± 0.10b		0.28 ± 0.10b		0.31 ± 0.10b
LR χ^2^*; df; P*		*0; 1; 1*						*7.71; 1; 0.005*		*5.85; 1; 0.016*		*50.96; 1; < 0.001*
Untreated	6	0.19 ± 0.07a		–		–	8	0.59 ± 0.14a	8	1.1 ± 0.14	8	2.28 ± 0.30a
Treated		0.03 ± 0.03b		–		–		0.09 ± 0.09b		0.69 ± 0.10		0.06 ± 0.06b
LR χ^2^*; df; P*		*3.96; 1; 0.046*						*12.97; 1; < 0.001*		*2.99; 1; 0.084*		*85.529; 1; < 0.001*
Untreated	8	0.41 ± 0.14	10	0.94 ± 0.16a	10	1.19 ± 0.32a	10	0.59 ± 0.09a	10	1.75 ± 0.20	10	1.06 ± 0.31a
Treated		0.31 ± 0.12		0.22 ± 0.09b		0.66 ± 0.37b		0.16 ± 0.06b		1.34 ± 0.17		0.06 ± 0.06b
LR χ^2^*; df; P*		*0.39; 1; 0.531*		*15.4; 1; < 0.001*		*4.97; 1; 0.026*		*8.71; 1; 0.003*		*1.71; 1; 0.191*		*34.46; 1; < 0.001*
Untreated	10	0.56 ± 0.16	12	0.47 ± 0.15a	12	0.97 ± 0.19a	12	0.25 ± 0.08	12	0.78 ± 0.45		
Treated		0.47 ± 0.13		0.19 ± 0.07b		0.06 ± 0.04b		0.16 ± 0.07		0.41 ± 0.12		
LR χ^2^*; df; P*		*0.2; 1; 0.654*		*3.98; 1; 0.046*		*30.66; 1; < 0.001*		*0.7; 1; 0.403*		*1.29; 1; 0.255*		
Untreated	12	0.41 ± 0.11	14	0.25 ± 0.09a	14	0.19 ± 0.08	14	0.00 ± 0.00	14	0.00 ± 0.00		
Treated		0.28 ± 0.09		0.03 ± 0.03b		0.06 ± 0.04		0.03 ± 0.03		0.00 ± 0.00		
LR χ^2^*; df; P*		*0.77; 1; 0.380*		*6.2; 1; 0.013*		*2.09; 1; 0.148*		*1.37; 1; 0.239*		*0; 1; 1*		
Untreated	14	0.19 ± 0.07										
Treated		0.19 ± 0.09										
LR χ^2^*; df; P*		*0; 1; 1*										

A comparison is made within a year and the same WAP, and between the untreated and the treated plots, values followed by the same letter are not statistically different with the generalized linear model with a Poisson or quasi-Poisson error distribution (*P* = 0.05). WAP, weeks after planting.

At Haut Katanga, when considering sites, the Kanyameshi site recorded 0.35 ± 0.05 and 0.27 ± 0.04 larvae/plant for the untreated and treated plots, respectively (LR*χ*^2^ = 1.90; *df* = 1; *P* = 0.168), while Kipopo recorded the highest larval abundance in the untreated (0.67 ± 0.08 larvae/plant) compared to treated plots (0.20 ± 0.06 larvae/plant) (LR*χ*^2^ = 49.98; *df* = 1; *P* < 0.001). Similarly, in Sud Kivu, the untreated at Bishibiru (0.39 ± 0.05 larvae/plant) and Mulungu (1.38 ± 0.12 larvae/plant) sites recorded significantly higher larval abundance than in treated plots at Bishibiru (0.11 ± 0.03 larvae/plant) and at Mulungu (0.39 ± 0.04 larvae/plant), respectively (Bishibiru: LR*χ*^2^ = 25.60; *df* = 1; *P* < 0.001; Mulungu: LR*χ*^2^ = 72.02; *df* = 1; *P* < 0.001).

### Damage Severity

*Global analyses*. In Cameroon, the damage severity was significantly affected by treatment (*P* < 0.001) and the location (*P* < 0.001), as well as the interaction between the treatment and the location (*P* < 0.001). The extent of the damage severity reduction by the pesticide depended on the location. Generally, Avebe and Memiam recorded higher damage severity, and, therefore, the effect of the pesticide in those locations was more conspicuous (Table 9).

**Table 9.** Fall armyworm damage severity comparison between Emamectin benzoate-treated and untreated maize in 4 experimental sites in the humid forest of Cameroon over 2 cropping seasons

Agroecology	Location/Field	Treatments	Damage severity (%)	*Z*, *P*
Forest (Mbalmayo)	Avebe	Untreated	32.7 ± 1.13 b	112.35;<0.001
		Treated	6.96 ± 0.59 a	
	Memiam	Untreated	20.18 ± 0.97 b	80.44;<0.001
		Treated	5.81 ± 0.59 a	
Mean Mbalmayo			16.43 ± 0.46	
Transitional zone (Batchenga)	Njie	Untreated	3.97 ± 0.39 a	32.71;<0.001
		Treated	1.33 ± 0.18 b	
	Okongolo	Untreated	2.34 ± 0.27 b	12.45;<0.001
		Treated	1.51 ± 0.25 a	
Mean Batchenga			2.29 ± 0.46	

Means comparison between the treated and untreated plots was done within each location.

Similarly to Cameroon, in the Democratic Republic of Congo, the damage severity was significantly affected by treatment (*P* < 0.001) and the location (*P* < 0.001), as well as the interaction between the treatment and the location (*P* < 0.001). The damage reduction level achieved by the pesticide application varied according to the location. The damage severity was generally higher in Kanyameshi and Kipopo (Haut Katanga) than in Bishibiru and Mulungu (Sud Kivu), leading to a more conspicuous effect of the pesticide application in the Huat Katanga (Table 10).

**Table 10.** Fall armyworm damage severity comparison between Emamectin benzoate-treated and untreated maize in 4 experimental sites in the humid forest/humid savannah of DR Congo over 2 cropping seasons.

Province	Location	Treatments	Leaf damage (%)	*Z*, *P*
Haut Katanga	Kanyameshi	Untreated	32.25 ± 1.06 b	3.49;<0.001
		Treated	16.45 ± 0.76 a	
	Kipopo	Untreated	37.35 ± 1.01 b	10.72;<0.001
		Treated	17.48 ± 0.66 a	
Mean Haut Katanga			26.26 ± 0.49 b	
Sud Kivu	Bishibiru	Untreated	13.76 ± 0.66 a	15.470;<0.001
		Treated	11.48 ± 0.43 b	
	Mulungu	Untreated	27.57 ± 0.67 a	13.501;<0.001
		Treated	17.53 ± 0.45 b	
Mean Sud Kivu			19.01 ± 0.32 a	

Means comparison between the treated and untreated plots was done within each location.

*Site-specific analysis*. The damage severity was constantly very low. When combining the untreated and treated plots across all experimental sites, 84.38% of the cases had less than 20% severity (scale 1), with 75.00% having less than 10% severity and even 60.94% having less than 5% damage severity. In the untreated plots, 75.00% of the cases had less than 20% severity (scale 1), with 62.50% having less than 10% severity and 46.88% having less than 5% damage severity. The low severity is even more pronounced in treated plots where 93.75% of the cases had less than 20% severity (scale 1), with 87.50% having less than 10% severity and even 75.00% having less than 5% damage severity. At the Batchenga site, 96.88% of the cases had less than 10% damage severity (Table 5). As a result, in 62.50% of the cases, the severity in the untreated plots was significantly higher than in the treated plots (*W* = 8677; *P* < 0.001—*W* = 6670.5; *P* = 0.028) (Table 11).

**Table 11.** Fall armyworm damage severity comparison between Emamectin benzoate-treated and untreated maize in 4 experimental sites at different sampling dates in the humid forest of Cameroon over 2 cropping seasons

WAP	Treatments	Mbalmayo district				Batchenga district			
		Memiam 2020	Memiam 2021	Avebe 2020	Avebe 2021	Ndjie 2020	Ndjie 2021	Okongolo 2020	Okongolo 2021
4	Untreated	30.61 ± 3.00a	28.39 ± 3.57	13.25 ± 2.31	25.67 ± 3.00	0.06 ± 0.05	6.68 ± 1.29a	1.86 ± 0.69	3.62 ± 0.99
	Treated	20.9 ± 2.86b	16.74 ± 2.79	16.35 ± 2.42	20.71 ± 2.89	0.4 ± 0.36	6.17 ± 1.06b	3.72 ± 1.34	1.06 ± 0.32
	W; p	*8039.5; 0.012*	*7078; 0.051*	*5331.5; 0.167*	*6865.5; 0.179*	*6270.5; 0.993*	*6304.5; 0.028*	*5849.5; 0.323*	*6666;0.204*
6	Untreated	13.01 ± 2.12a	13.01 ± 2.12a	31.81 ± 2.91a	64.96 ± 2.69a	3.84 ± 1.36	11.24 ± 1.9a	3.84 ± 1.36	4.53 ± 0.86
	Treated	4.25 ± 1.32b	4.25 ± 1.32 b	3.49 ± 0.97b	11.61 ± 1.97b	0.59 ± 0.15	1.87 ± 0.43b	0.59 ± 0.15	4.39 ± 1.06
	W; p	*8677; < 0.001*	*8677; < 0.001*	*10408; < 0.001*	*12510; < 0.001*	*6522; 0.436*	*7883; < 0.001*	*6522; 0.436*	*6725; 1; 0.274*
8	Untreated	8.43 ± 1.6a	28.42 ± 2.73a	9.60 ± 1.50a	63.17 ± 2.59a	1.47 ± 0.92a	5.91 ± 0.98a	0.4 ± 0.16	3.81 ± 1.07a
	Treated	0.35 ± 0.17b	1.18 ± 0.29b	0.64 ± 0.35b	1.95 ± 0.55b	0.06 ± 0.05b	1.06 ± 0.51b	0.24 ± 0.12	0.73 ± 0.22b
	W; p	*8351.5; < 0.001*	*10424; < 0.001*	*9135; < 0.001*	*13325; < 0.001*	*6670.5; 0.028*	*8551; < 0.001*	*6388; 0.542*	*7216; 0.005*
11	Untreated	4.53 ± 1.23a	8.54 ± 1.28a	4.51 ± 1.14a	43.54 ± 2.69a	0.39 ± 0.21	2.17 ± 0.33 a	0.2 ± 0.09 a	1.63 ± 0.76 a
	Treated	0.34 ± 0.23b	0.22 ± 0.14b	1.94 ± 0.66b	0.25 ± 0.12b	0.17 ± 0.11	0.30 ± 0.13 b	0.04 ± 0.04 a	0.04 ± 0.04 b
	W; P	*7688.5; < 0.001*	*9214; < 0.001*	*6795.5; < 0.001*	*12916; < 0.001*	*6493.5; 0.203*	*8078.5; < 0.001*	*6551; 0.057*	*7058.5; < 0.001*

A comparison is made within a year, and the same sampling is used, and between the untreated and the treated, values followed by the same letter are not statistically different from Wilcoxon (*P* = 0.05). WAP, weeks after planting.

At Mbalmayo, when combining both years for each site, the Avebe site recorded 32.70% ± 1.13 and 6.96% ± 0.60% damage severity for the untreated and treated plots, respectively (*W* = 61; *df* = 1; *P* < 0.001), while Memiam recorded 20.18% ± 0.97% and 5.81% ± 0.59% damage severity, respectively (*W* = 56, *df* = 1; *P* < 0.001). At Batchenga in the same conditions, the Ndjie site recorded 3.97% ± 0.39% and 1.33% ± 0.18% damage severity for the untreated and treated plots, respectively (*W* = 45; *df* = 1; *P* < 0.001), while Okongolo recorded 2.34% ± 0.27% and 1.51% ± 0.25% damage severity, respectively (*W* = 42; *df* = 1; *P* < 0.001).

In the Democratic Republic of Congo, was significantly affected by treatment (LR*χ*^2^ = 663.84; *df* = 1; *P* < 0.001), the location (LR*χ*^2^ = 103.36; *df* = 1; *P* < 0.001), the season (LR*χ*^2^ = 409.31; *df* = 1; *P* < 0.001), as well as the interaction between the treatment and the season (LR*χ*^2^ = 123.21; *df* = 1; *P* < 0.001). The interaction between the treatment and the location was not significant (LR*χ*^2^ = 0.53; *df* = 1; *P* = 0.466). Higher damage severity rates compared to Cameroon were recorded, with only 45.00%, 11.67%, and 1.67% of cases below 20%, 10%, and 5% damage severity, respectively (Table 6). In the treated plots, however, 66.67% of the cases recorded damage severity below 20% compared to just 23.33% of cases for the untreated. Consequently, in 80.00% of the cases, the damage severity in the untreated plots was significantly higher than the treated (*W* = 4807; *P* < 0.001—*W* = 3770.5; *P* = 0.026) (Table 12).

**Table 12.** Fall armyworm damage severity comparison between Emamectin benzoate-treated and untreated maize in 4 experimental sites at different sampling dates in the humid forest/humid savannah of DR Congo over 2 cropping seasons

Treatments	Haut Katanga Province						Sud Kivu Province					
	Kanyameshi 2020		Kipopo 2020		Kipopo 2021		Bishibiru 2020		Mulungu 2020		Mulungu 2021	
	WAP	Severity (%)	WAP	Severity (%)	WAP	Severity (%)	WAP	Severity (%)	WAP	Severity (%)	WAP	Severity (%)
Untreated	4	34.71 ± 2.60a	6	24.01 ± 3.37a	6	27.61 ± 1.78	6	6.64 ± 0.84a	6	18.00 ± 1.61a	4	20.19 ± 1.63
Treated		14.62 ± 1.61b		4.56 ± 1.01b		27.46 ± 2.09		11.74 ± 0.72b		13.87 ± 1.18b		21.59 ± 1.50
W; p		*4807; < 0.001*		*4114.5; < 0.001*		*3267.5; 0.810*		*1846.5; < 0.001*		*3672.5; 0.02*		*2981.5; 0.42*
Untreated	6	34.71 ± 2.60a	8	34.54 ± 2.92a	8	33.25 ± 2.24	8	16.86 ± 1.50a	8	22.5 ± 1.61a	8	35.74 ± 2.40a
Treated		14.62 ± 1.61b		12.38 ± 1.20b		33.25 ± 2.43		11.74 ± 0.72b		17.49 ± 1.28b		22.62 ± 1.37b
W; p		*4807;<0.001*		*4767;<0.001*		*3241.5; 0.884*		*3719; 0.008*		*3770.5; 0.026*		*4339.5; < 0.001*
Untreated	8	40.59 ± 2.58a	10	31.32 ± 3.35a	10	60.37 ± 2.81a	10	18.5 ± 1.57	10	27.00 ± 1.70a	8	39.75 ± 2.10a
Treated		15.64 ± 1.58b		5.35 ± 1.66b		29.5 ± 1.98b		14.24 ± 0.99		20.24 ± 1.50b		13.99 ± 0.98b
W; p		*5174.5; < 0.001*		*4845.5; < 0.001*		*5278.5; < 0.001*		*3626; 0.054*		*3980; 0.004*		*5552.5; < 0.001*
Untreated	10	38.83 ± 2.31a	12	24.77 ± 2.22a	12	56.72 ± 3.56a	12	19.00 ± 1.66a	12	30.00 ± 1.70a	10	38.5 ± 1.87a
Treated		16.1 ± 1.97b		6.70 ± 1.24b		23.87 ± 1.79b		13.71 ± 1.11b		21.58 ± 1.69b		10.86 ± 0.49b
W; p		*5020; < 0.001*		*4930; < 0.001*		*4994; < 0.001*		*3779; 0.011*		*4158; < 0.001*		*5875; < 0.001*
Untreated	12	36.05 ± 2.52a	14	28.78 ± 2.31a	14	52.11 ± 3.44a	14	7.80 ± 0.92	14	16.50 ± 1.03		
Treated		21.23 ± 2.079b		7.57 ± 1.42b		24.19 ± 1.97b		5.97 ± 0.91		15.57 ± 1.20		
W; p		*4379.5; < 0.001*		*5065; 0.001*		*4842.5; < 0.001*		*3668; 0.074*		*3412; 0.392*		
Untreated	14	35.83 ± 2.42a										
Treated		17.59 ± 2.27b										
*W; P*		4678; < 0.001										

A comparison is made within a year, and the same sampling is used, and between the untreated and the treated, values followed by the same letter are not statistically different from Wilcoxon (*P* = 0.05). WAP, weeks after planting.

At Haut Katanga, when considering sites, the Kanyameshi site recorded 32.25% ± 1.60% and 16.44% ± 0.76% damage severity for the untreated and treated plots, respectively (*W* = 16; *df* = 1; *P* < 0.001), while Kipopo recorded 37.35% ± 1.01% and 17.48% ± 0.66% damage severity, respectively (*W* = 45; *df* = 1; *P* < 0.001). At Sud Kivu, the Bishibiru site recorded 13.76% ± 0.66% and 11.48% ± 0.43% damage severity for the untreated and treated plots, respectively (*W* = 83; *df* = 1; *P* = 0.281), while the Mulungu site recorded 27.57% ± 0.67% and 17.53% ± 0.45% damage severity, respectively (*W* = 34; *df* = 1; *P* < 0.001).

*Yield*. Despite the 62.50% and 56.67% success rates in reductions of FAW damage incidence as well as the 62.50% and 80.00% success rates in reductions of FAW damage severity in Cameroon and the Democratic Republic of Congo, respectively, pesticide application against fall armyworm did not result in a significant effect on yield in any of the seasons, locations, or countries during the present study. In Ndjie in 2020, the yield was 4.037 ± 0.78 t/ha on the treated plots and 3.587 ± 0.72 t/ha on the untreated plots; in 2021, it was 4.47 ± 0.22 t/ha and 4.19 ± 0.24 t/ha on the treated and untreated plots, respectively. The yield in Okongolo in 2020 was 4.84 ± 0.53 t/ha and 4.42 ± 0.47 t/ha on the treated and untread plots, respectively. In Avebe in 2020, the yield was 3.47 ± 0.03 t/ha on the treated plots and 3.35 ± 0.14 t/ha on the untreated plots; in 2021, it was 4.97 ± 0.09 t/ha on the treated plots and 4.54 ± 0.15 t/ha on the untreated plots. In Memiam in 2020, the yield was 3.87 ± 0.27 t/ha and 3.77 ± 0.25 t/ha on the treated and untreated plots, respectively; in 2021, it was 4.46 ± 0.39 t/ha and 4.26 ± 0.39 t/ha on the treated and untreated plots, respectively.

In Kipopo, the yield on the treated plots in 2020 was 7.08 ± 0.51 t/ha and on the untreated plots was 6.06 ± 0.38 t/ha; in 2021, it was 2.05 ± 0.16 t/ha on the treated plots and 1.85 ± 0.12 t/ha on the untreated plots. In Kanyameshi in 2020, it was 5.97 ± 0.27 t/ha on the treated plots and 5.52 ± 0.16 t/ha on the untreated plots. In Mulungu in 2020, the yield was 5.51 ± 0.12 t/ha, while on the untreated plots, it was 5.09 ± 0.17 t/ha; in 2021, the yield was 4.9 ± 0.23 t/ha and 5.79 ± 0.34 t/ha on the treated and untreated plots, respectively. In Bishibiru in 2020, it was 5.58 ± 0.31 t/ha on treated and 5.79 ± 0.34 t/ha.

The yield losses rarely exceeded 10%, except for Ndjie (Cameroon) in 2020, where a yield loss of 11.97% ± 3.89% (Table 13) was recorded, and Mulungu in 2021 and Kipopo in 2021 (the Democratic Republic of Congo), where 10.93% ± 4.10% and 10.33% ± 7.55% were registered, respectively (Table 14).

**Table 13.** Effect of the treatments on the percentage of yield lost and the cost–benefit ratio in 4 experimental sites in the humid forest of Cameroon over 2 cropping seasons

Site	Years	Treatment	Yield loss ± SE (%)	Cost–benefit ratio
Memiam	2020	Untreated	2.61 ± 0.76	−0.70 ± 1.20
		Treated		
	2021	Untreated	4.65 ± 1.60	−2.51 ± 0.70
		Treated		
Avebe	2020	Untreated	3.34 ± 3.41	−1.46 ± 1.17
		Treated		
	2021	Untreated	8.53 ± 4.20	−1.83 ± 1.97
		Treated		
Ndjie	2020	Untreated	11.97 ± 3.89	−1.17 ± 1.10
		Treated		
	2021	Untreated	6.05 ± 3.63	0.72 ± 1.86
		Treated		
Okongolo	2020	Untreated	6.41 ± 1.10	0.16 ± 2.30
		Treated		
	2021	Untreated	−5.23 ± 4.93	−2.85 ± 1.83
		Treated		

**Table 14.** Effect of the treatments on the percentage of yield lost and the cost–benefit ratio in 4 experimental sites in the humid forest/humid savannah of DR Congo over 2 cropping seasons

Site	Years	Treatment	Yield loss ± SE (%)	Cost–benefit ratio
Mulungu	2020	Untreated	7.59 **± **2.11	0.72 ± 0.09
		Treated		
	2021	Untreated	10.93 **± **4.10	1.09 ± 0.78
		Treated		
Bishibiru	2020	Untreated	−3.97 **± **2.98	−2.67 ± 1.30
		Treated		
Kanyameshi	2020	Untreated	6.77 **± **4.45	−0.98 ± 1.11
		Treated		
Kipopo	2020	Untreated	10.33 **± **7.55	−1.69 ± 1.64
		Treated		
	2021	Untreated	8.61 **± **1.71	−0.85 ± 0.97
		Treated		

*Cost–benefit analysis*. Similar to what was obtained for the yields, the farm gate revenues were similar between untreated and treated plots for all sites, all seasons, and countries. In Cameroon, farm gate revenues ranged from 1210.92 ± 52.99 USD/ha to 1796.18 ± 34.53 USD/ha. It ranged from 416.81 ± 28.15 USD/ha to 1593.37 ± 115.29 USD/ha in the Democratic Republic of Congo. Similarly, when the opportunity cost of the pesticide application is added to the equation, the net return does not significantly differ between the untreated and treated plots for all sites, all seasons, and countries. In Cameroon, the net return ranged from 1129.17 ± 52.99 USD/ha to 1673.65 ± 34.53 USD/ha, while in the Democratic Republic of Congo, it varied from 326.02 ± 54.96 USD/ha to 1269.417 ± 126.82 USD/ha.

The cost–benefit ratios of treating the maize field with emamectin benzoate are presented in Tables 13 and 14 for Cameroon and the Democratic Republic of Congo. In Cameroon, the highest ratio was 0.72 ± 1.86, and the lowest was −2.85 ± 1.83, while in the Democratic Republic of Congo, the highest was 1.09 ± 0.78, and the lowest was −2.67 ± 1.30. In Cameroon (Table 13), yield losses went up to 11.97%, but over the 8 cases presented in that table, the cost–benefit ratios were around 1 or below 1. The case is similar in the Democratic Republic of Congo (Table 14), i.e., the benefits of controlling fall armyworm did not cover twice the cost of the pesticide application and, in some cases, did not even cover it, or the application led to a loss.

## Discussion

The fall armyworm invasion in Africa has caused panic among stakeholders (Otim et al. 2021). The damage is unique and usually appears devastating, and the combination of farmers’ feelings and quick political responses has led to inappropriate responses to the pest, including the use of highly toxic pesticides (FAO 2018b), putting farmers, maize, the environment, and even livestock at risk of contamination (Carvalho et al. 2013, Harrison et al. 2019). Desperate responses to fall armyworm infestations in Africa may be due to a lack of research into management approaches. There is little understanding of how fall armyworm damage affects yields in different African agroecologies, as well as the economic benefits of intervention. Furthermore, studies have shown that many farmers are unaware of, or simply ignore, the potential dangers of insecticides to human health and nontarget insects (Kansiime et al. 2019, Abro et al. 2021). Therefore, any intervention, especially one based on insecticides, needs to be carefully considered. Most published yield losses due to fall armyworm on the African continent since the pest’s invasion are mainly based on questionnaires and interviews with farmers (Kansiime et al. 2019, Kumela et al. 2019, De Groote et al. 2020, Koffi et al. 2020, Andrew et al. 2023), which do not distinguish between actual yield losses and the frequency and severity of damage as perceived by farmers. Fall armyworm is a voracious phytophagous pest whose activity in the field can cause panic. Where crop losses have been reported through farmer surveys, yield losses due to fall armyworm may be overestimated, with yield losses being more than double those reported in experimental trials (Overton et al. 2021). The aim of the present study was, therefore, to provide biophysical data collected in a total of 14 site seasons over 2 consecutive years under humid tropical forest conditions in Cameroon and the Democratic Republic of Congo.

In Cameroon, damage incidence varied by site and season. In Memiam and Avebe, characterized by forest and relatively higher fall armyworm damage incidence and severity, chemical treatment had a significant effect on damage incidence, while in Ndjie (transitional forest savannah with lower fall armyworm damage incidence) the effect was only significant in 2021, and in Okongolo (same conditions as Ndjie) the effect of spraying was not significant. Similarly, in the Democratic Republic of Congo, the effect of spraying was significant except at Kipopo in 2021 (semi-humid and savannah zones) and Mulungu (humid forest zone) in 2021, where a significant effect on damage incidence was only observed at late sampling dates when the effect is expected to be less compared to earlier stages. High incidences of up to 90% and 100% were recorded in Cameroon and the Democratic Republic of Congo, respectively. Although the use of insecticides had a significant effect on the incidence of damage, it did not give us any information on the potential impact of the pest on the crop. The incidence of damage only gave us information on the distribution of the pest in the field and how often it fed on at least one leaf of the sampled plants. Currently, the decision to intervene in farmers’ fields is largely based on the level of damage incidence (Prasanna et al. 2012, 2018, FAO and PPD 2020, FAO and DPPQS 2021). On its own, it does not represent an adequate reference to develop a relevant decision tool for management intervention. In the current study, although a high incidence was achieved in certain seasons, it was not translated into yield losses.

Larval abundance was generally not significantly affected by insecticide application in Cameroon when considering each site due to the very low number of larvae recorded. In the Democratic Republic of Congo, where a significant effect was observed in most of the cropping seasons and sampling dates, even a mean abundance of 3 larvae per plant did not result in a significant difference in yield. However, this was the highest larval density observed in maize in this study. Fall armyworm is a boom-or-bust insect. Most of the time, the pressure is low or moderate (Barfield et al. 1980, Fuxa 1982, Feed the Future 2018, IAPPS 2022). Occasionally, the system becomes unbalanced, and farmers experience an outbreak that can affect yields (Fuxa 1982, Feed the Future 2018, IAPPS 2022). However, there is a general tendency to overestimate the impact of fall armyworms across Africa, as shown by Harrison et al. (2022). In the present study in both countries, we observed during the field trials that larval abundance is usually low and tends to disappear during the reproductive stage of the plant. Therefore, little or no impact on the maize cob is observed, resulting in no direct grain losses. Larval densities per plant were mostly less than 1 larva/plant at the different study sites. While maize varieties produced high biomass in the studied sites under tropical humid agroecology, the recorded pest pressure was too low to damage the leaves to a detrimental level to the crop or to reduce the photosynthetic leaf area of the crop. In fact, several studies have shown that heavy rainfall has a negative impact on the development of fall armyworms. According to Sims (2008), increasing the amount of simulated rainfall significantly reduced the emergence of fall armyworm adults. Heavy rainfall reduces adult emergence by trapping moths in their pupation tunnel, and in more fragile soils, rainfall causes the soil to collapse (Sims 2008). Similarly, Silvain and Ti-A-Hing (1985) reported that larval populations were influenced by the amount of rainfall 3 wk prior to field surveys. Studies conducted by Ghidiu and Drake (1989) showed that leaf-feeding damage, the percentage of damaged tassels, and the percentage of damaged ears should increase as the number of fall armyworm larvae per plant increases. In the current agroecology, a very low density of fall armyworm larvae per plant was recorded, with negligible damage to ears. The impact on yield is high if there is seedling death, a damaged growing point, or significant defoliation at critical growth stages. In addition, feeding damage to tassels and cobs during pollination and grain filling will affect yield and grain quality (Anjorin et al. 2022). These facts were not or rarely observed in the agroecology where the current studies were conducted. This is also supported by studies conducted by Abang et al. (2021) in humid forest conditions in Cameroon, which showed mutual exclusion between fall armyworm and stemborers, with fall armyworm present at tender ages but giving way to stemborers from the tasseling stage where the leaves become harder.

Damage severity was often significantly influenced by the insecticide application. However, the significant difference in damage severity did not result in a significant difference in yield, even where high damage severity was recorded. While no data are available on the relationship between leaf damage and yield loss under African growing conditions, most of the literature from North and South America indicates no to poor correlations between the level of fall armyworm infestation, leaf damage, and actual yield loss (van den Berg et al. 2021). As with many agricultural arthropod pests, the economic impact of fall armyworms is complicated by interactions between host plant species, plant growth stage, pest life stages, and environmental context (Buntin 1986). Previous studies have shown that plants with adequate nutrition are stronger, healthier, and generally better able to compensate for pest damage than those with nutritional deficiencies (Maxwell and Jennings 1980). To assess the damage-yield relationship for fall armyworm, Blanco et al. (2022) manually removed 0%, 33%, and 66% of the foliage when maize had 1–2 (V1–V2) and 3–4 (V3–V4) fully developed leaves. The amount of defoliation did not reduce the yield potential of maize compared to nondefoliated plants, regardless of the timing of defoliation: V1–V2 or V3–V4. However, fall armyworm-induced leaf damage has been reported to cause significant yield losses at certain plant stages (Chisonga et al. 2023). Fertilization of defoliated plants yielded significantly more grain than unfertilized plants, and these clear results showed that smallholder maize farmers who can afford to invest in either fertilizer or insecticide will benefit more from the former.

They concluded that young maize plants can compensate for large amounts of defoliation without reducing yield. Therefore, in tropical humid agroecology, where rainfall can negatively affect larval abundance, high damage severity should not justify pesticide application under good agricultural practices. Studies conducted by Murúa et al. (2006) in northwestern Argentina showed that temperature and rainfall were the climatic factors that significantly affected fall armyworm density. In addition, foliar damage to maize may appear severe but does not necessarily translate into high grain yield losses (Hruska 2019). Hruska (2019) reported that the US Department of Agriculture published the results of NCIS research in the Corn Loss Adjustment Standards Handbook in 2013, noting that fall armyworm defoliation of up to 70% at the 12-leaf stage could result in only about 15% grain yield loss. Fall armyworm defoliation in maize rarely exceeds 50% (Hruska 2019). Studies conducted by Osae et al. (2022) in the coastal savannah agroecological zone of Ghana showed that fall armyworm infestation had no effect on maize grain yield on the fresh cob and dry grain yield shelled from the dry cob. A study of the minimum severity of photosynthetic leaf area damage that could affect maize under tropical humid agroecology and good agricultural practices is warranted.

The nonsignificant effect of damage severity on yield was also supported by the level of yield losses recorded. In fact, yield losses rarely exceeded 10% (11.97% ± 3.89% in Cameroon and 10.93% ± 4.10% in the Democratic Republic of Congo). In a study conducted by Baudron et al. (2019), the impact of fall armyworm damage on yield was estimated at 11.57%. They stated that previous studies may have overestimated yield losses due to fall armyworm. The same situation was reported by Harrison et al. (2022), who suggested that the impact of *S. frugiperda* may have been overestimated in many locations in sub-Saharan Africa. It is important to note that in the present study, and generally in humid forest agroecological conditions, the maize varieties produced are characterized by high biomass production, and robust studies combining the photosynthetic threshold for optimal yield production with the actual leaf area lost to fall armyworm in the tropical humid forest agroecologies are warranted.

There was no significant difference in the net return between untreated and treated plots. Similarly, the cost–benefit ratio showed that the additional benefit of pesticide application rarely compensated for the cost of pesticide application. This is the most critical part that all IPM options should demonstrate before dissemination. There is no need to treat a field, even if there is defoliation or no added value for farmers compared to those who have not intervened. And if these control measures are chemical pesticides, this is not only economically counterproductive but also carries human and environmental risks, particularly in smallholder systems where farmers rarely use full protective measures when applying pesticides.

This study showed that the use of chemical pesticides can effectively reduce the number of larvae and the amount of damage they cause. However, this method does not have a significant impact on crop yield or the net return of farmers in the agroecological conditions of the tropical humid forest in Central Africa. Farmers in such agroecologies should refrain from using pesticides and instead rely on the adoption of good agricultural practices that contribute to significant yield increases. In the study areas, 2 applications of chemical pesticides 2 wk apart were recommended, and this was used in the present study. Based on the number of sites and years in which the present study was conducted, we recommend that in humid tropical forest agroecology, the number of pesticide applications during a cropping season should be at least reduced and even dropped to avoid pesticide wastage, increased production costs, and environmental and human health concerns. In a study conducted by Osae et al. (2022), 2 applications of Ampligo (Chlorantraniliprole and Lambda-Cyhalothrine) at 200 ml/ha, 1 wk apart, are sufficient to maintain maize for the entire cropping season in the Coastal Savannah Agroecological Zone of Ghana. Incidence-based action thresholds (10%–30%; 30%–50%; 10%–30% for Early Whorl Stage, Late Whorl Stage, and Tassel & Silk Stage, respectively) are not recommendable in humid tropical agroecologies. Control decision tools such as action thresholds or economic thresholds for fall armyworms in such ecologies need to be established based on leaf damage and/or larval abundance. The present study, therefore, calls for a change in the approach to fall armyworm management, which is currently characterized by the scaling up and promotion of interventions against the pest without consideration of local agroecological conditions. In tropical humid forest agroecologies in general, interventions, whether chemical or biopesticide, may not be economically viable for farmers.

## Data Availability

Data from this study is available from the International Institute of Tropical Agriculture. https://doi.org/10.25502/0R89-XA94/D (Agbodzavu et al. 2024).

## References

[CIT0001] Abang AF , Fotso Kuate A, Nanga Nanga S, Okomo Esi RM, Ndemah R, Masso C, Fiaboe KKM, Hanna R. Spatio-temporal partitioning and sharing of parasitoids by fall armyworm and maize stemborers in Cameroon. J Econ Entomol. 2021:145(1):55–64. 10.1111/jen.12827

[CIT0002] Abro Z , Kimathi E, de Groote H, Tefera T, Sevgan S, Niassy S, Kassie M. Socioeconomic and health impacts of fall armyworm in Ethiopia. PLoS One. 2021:16(11):e0257736. 10.1371/journal.pone.0257736 34735485 PMC8568106

[CIT0003] Feed the Future. Fall armyworm (FAW) management report: evaluating least toxic and cost-effective approaches to FAW management. Advance FAW management report 2018. USAID, ACDI/VOCA; 2018.

[CIT0004] Agbodzavu KM , Nanga Nang S, Abang AF, Fotso-Kuate A, Bamba Z, Masso C, Fiaboe KKM. Impact of Fall armyworm, *Spodoptera frugiperda* (Lepidoptera: Noctuidae), on maize yield in humid tropical zones of Central Africa [dataset]. International Institute of Tropical Agriculture; 2024. 10.25502/0R89-XA94/D PMC11318617 38768376

[CIT0005] Ahissou BR , Sawadogo WM, Bokonon-Ganta AH, Somda I, Verheggen F. Integrated pest management options for the fall armyworm *Spodoptera frugiperda* in West Africa: challenges and opportunities. A review. *Biotechnol**Agron**Soc**Environ.* 2021:25(3):192–207. 10.25518/1780-4507.19125. https://popups.uliege.be/1780-4507

[CIT0006] Andrew K , Michael OH, Tom W, Wee TT. Farmer perception of impacts of fall armyworm (*Spodoptera frugiperda* JE Smith) and transferability of its management practices in Uganda. CABI Agric Biosci. 2023:4:1–14.

[CIT0007] Anjorin FB , Odeyemi OO, Kareem KT. Fall armyworm (*Spodoptera frugiperda*) (JE Smith) (Lepidoptera: Noctuidae) infestation: maize yield depression and physiological basis of tolerance. J Plant Prot Res. 2022:62(1):12–21.

[CIT0008] Barfield CS , Stimac JL, Keller MA. Fall armyworm symposium: state-of-the-art for predicting damaging infestations of fall armyworm. Fla Entomol. 1980:63(4):364–375. 10.2307/3494520

[CIT0009] Bates D , Maechler M, Bolker B, Walker S. lme4: linear mixed-effects models using ‘Eigen’ and S4; 2023 [accessed 2024 May 13]. https://github.com/lme4/lme4/.

[CIT0010] Baudron F , Zaman-Allah MA, Chaipa I, Chari N, Chiwanda P. Understanding the factors influencing fall armyworm (*Spodoptera frugiperda* J.E. Smith) damage in African smallholder maize fields and quantifying its impact on yield. A case study in Eastern Zimbabwe. Crop Prot. 2019:120(June 2019):141–150.

[CIT0011] Blanco CA , Conover K, Hernandez G, Valentini G, Portilla M, Abel CA, Williams P, Nava-Camberos U, Hutchison WD, Dively GP. Grain yield is not impacted by early defoliation of maize: implications for fall armyworm action thresholds. Southwest Entomol. 2022:47(2):335–344.

[CIT0012] Blumenthal JM , Thompson WH. Estimating corn grain yields. Texas: Extension Publication; 2009. p. 6.

[CIT0013] Buntin GD. Fall armyworm symposium on plant resistance: a review of plant response to fall armyworm, *Spodoptera Frugiperda* (J. E. Smith), injury in selected field and forage crops. Fla Entomol. 1986:69(3):549–559. 10.2307/3495389

[CIT0014] Cairns JE , Chamberlin J, Rutsaert P, Voss RC, Ndhlela T, Magorokosho C. Challenges for sustainable maize production of smallholder farmers in sub-Saharan Africa. J Cereal Sci. 2021:101(September 2021):103274. 10.1016/j.jcs.2021.103274

[CIT0015] Carvalho RA , Omoto C, Field LM, Williamson MS, Bass C. Investigating the molecular mechanisms of organophosphate and Pyrethroid resistance in the fall armyworm *Spodoptera frugiperda*. PLoS One. 2013:8(4):e62268. 10.1371/journal.pone.0062268 23614047 PMC3629120

[CIT0016] Carvalho RPL. Danos, flutuação da população, controle e comportamento de Spodoptera frugiperda (Smith 1797) e suscetibilidade de diferentes genótipos de milho em condições de campo [Tese (Doutorado)]. São Paulo, Brazil: ESALQ/USP, Piracicaba; 1970.

[CIT0017] CBFP. The forests of the Congo Basin: a preliminary assessment. 2005. p. 39 [accessed 2024 May 10]. https://pfbc-cbfp.org/forests-2005.html.

[CIT0018] Chisonga C , Chipabika G, Sohati PH, Harrison RD. Understanding the impact of fall armyworm (*Spodoptera frugiperda* J. E. Smith) leaf damage on maize yields. PLoS One. 2023:18(6):e0279138. 10.1371/journal.pone.0279138 37307270 PMC10259777

[CIT0019] Day R , Abrahams P, Bateman M, Beale T, Clottey V, Cock M, Colmenarez Y, Corniani N, Early R, Godwin J, et al. Fall armyworm: impacts and implications for Africa. Outlooks Pest Manag. 2017:28(5):196–201. 10.1564/v28_oct_02

[CIT0020] De Groote H. Maize yield losses from stemborers in Kenya. Int J Trop Insect Sci. 2002:22(02):89–96. 10.1017/s1742758400015162

[CIT0021] De Groote H , Kimenju SC, Munyua B, Palmas S, Kassie M, Bruce A. Spread and impact of fall armyworm (*Spodoptera frugiperda* J.E. Smith) in maize production areas of Kenya. Agric Ecosyst Environ. 2020:292(April 2020):106804. 10.1016/j.agee.2019.106804 32308246 PMC7015277

[CIT0022] DeWasseige C , Tadoum M, Eba’a Attyi R, Doumenge C . The forest of Congo basin-forest and climate change. Weyrich Edition. Neufchâteau, Belgium: CIFOR Publications; 2015. ISBN 978-2-87489-355-1.

[CIT0023] Ekpa O , Palacios-Rojas N, Kruseman G, Fogliano V, Linnemann AR. Sub-Saharan African maize-based foods: technological perspectives to increase the food and nutrition security impacts of maize breeding programs. Global Food Secur. 2018:17(June 2018):48–56. 10.1016/j.gfs.2018.03.007

[CIT0024] FAO, DPPQS. Integrated pest management (IPM) farmer field school (FFS) – a guide for facilitators of FFS on maize with special emphasis on fall armyworm. New Delhi: FAO, DPPQS; 2021.

[CIT0025] FAO, PPD. Manual on integrated fall armyworm management. Yangon: FAO; 2020.

[CIT0027] FAO. Integrated management of the fall armyworm on maize: a guide for farmer field schools in Africa. Rome, Italy: FAO; 2018b.

[CIT0028] FAO. The Republic of Namibia – fall armyworm impact and needs assessment – 2018: La FAO en situations d’urgence. Rome, Italy: FAO; 2018a.

[CIT0029] FAOSTAT. Food and Agriculture Organization of the United Nations (FAO). FAOSTAT Database. 2016. http://faostat.fao.org/site/291/default.aspx

[CIT0030] Fox J , Weisberg S. An R companion to applied regression. 3rd ed. Thousand Oaks (CA): Sage; 2019. https://socialsciences.mcmaster.ca/jfox/Books/Companion/.

[CIT0031] Fuxa JR. Prevalence of viral infections in populations of fall armyworm, *Spodoptera frugiperda*, in Southeastern Louisiana. Environ Entomol. 1982:11(1):239–242. 10.1093/ee/11.1.239

[CIT0032] Ghidiu GM , Drake GE. Fall armyworm (Lepidoptera: Noctuidae) damage relative to infestation level and stage of sweet corn development. J Econ Entomol. 1989:82(4):1197–1200. 10.1093/jee/82.4.1197

[CIT0033] Goergen G , Kumar PL, Sankung SB, Togola A, Tamò M. First report of outbreaks of the fall armyworm *Spodoptera frugiperda* (J. E. Smith) (Lepidoptera, Noctuidae), a new alien invasive pest in West and Central Africa. PLoS One. 2016:11(10):e0165632. 10.1371/journal.pone.0165632 27788251 PMC5082806

[CIT0034] Grote U , Fasse A, Nguyen TT, Erenstein O. Food security and the dynamics of wheat and maize value chains in Africa and Asia. Front Sustain Food Syst. 2021:4:317.

[CIT0035] Hailu G , Niassy S, Bässler T, Ochatum N, Studer C, Salifu D, Agbodzavu MK, Khan ZR, Midega C, Subramanian S. Could fall armyworm, *Spodoptera frugiperda* (J. E. Smith) invasion in Africa contribute to the displacement of cereal stemborers in maize and sorghum cropping systems? Int J Trop Insect Sci. 2021:41(2):1753–1762. 10.1007/s42690-020-00381-8

[CIT0036] Harrison R , Banda J, Chipabika G, Chisonga C, Katema C, Mabote Ndalamei D, Nyirenda S, Tembo H. Low impact of fall armyworm (*Spodoptera frugiperda* Smith) (Lepidoptera: Noctuidae) across smallholder fields in Malawi and Zambia. J Econ Entomol. 2022:115(6):1783–1789. 10.1093/jee/toac113 36515111 PMC9748589

[CIT0037] Harrison RD , Thierfelder C, Baudron F, Chinwada P, Midega C, Schaffner U, van den Berg J. Agro-ecological options for fall armyworm (*Spodoptera frugiperda* J.E. Smith) management: providing low-cost, smallholder-friendly solutions to an invasive pest. J Environ Manage. 2019:243(August 2019):318–330. 10.1016/j.jenvman.2019.05.011 31102899

[CIT0038] Hruska AJ. Fall armyworm (Spodoptera frugiperda) management by smallholders. CAB Rev Perspect Agric Vet. 2019:14(43):1–11.

[CIT0039] IAPPS. Fall armyworm in USA: this year was a bust. Sex pheromones involved? 2022 [accessed 2023 Aug 4]. https://iapps2010.me/2022/08/24/fall-armyworm-in-usa-this-year-was-a-bust-sex-pheromones-involved/.

[CIT0040] Kansiime MK , Mugambi I, Rwomushana I, Nunda W, Lamontagne-Godwin J, Rware H, Phiri NA, Chipabika G, Ndlovu M, Day R. Farmer perception of fall armyworm (*Spodoptera frugiperda* J.E. Smith) and farm-level management practices in Zambia. Pest Manag Sci. 2019:75(10):2840–2850. 10.1002/ps.5504 31148397 PMC6771660

[CIT0041] Koffi D , Kyerematen R, Eziah VY, Osei-Mensah YO, Afreh-Nuamah K, Aboagye E, Osae M, Meagher RL. Assessment of impacts of fall armyworm, *Spodoptera frugiperda* (Lepidoptera: Noctuidae) on maize production in Ghana. J Integr Pest Manag. 2020:11(1):20–21.

[CIT0042] Kumela T , Simiyu J, Sisay B, Likhayo P, Mendesil E, Gohole L, Tefera T. Farmers’ knowledge, perceptions, and management practices of the new invasive pest, fall armyworm (*Spodoptera frugiperda*) in Ethiopia and Kenya. Int J Pest Manag. 2019:65(1):1–9. 10.1080/09670874.2017.1423129

[CIT0043] Lenth R. emmeans: Estimated marginal means, aka least-squares means. R package version 1.8.7; 2023 [accessed 2023 March 28]. https://CRAN.R-project.org/package=emmeans.

[CIT0044] Magdy AM , Osman ZA, Ahmed BS, Ahmed EM, Abou-gleel AA. Assessment of maize yield loss to determine economic injury levels (Eils) due to the infestation by stem borers with insecticidal control under the Egyptian conditions. Alex Sci Exch. 2016:37(4):729–737.

[CIT0045] MAPA. Projeções do agronegocio. In: Brasil 2018/19 a 2028/29 Projeções de Longo Prazo; Brasilia; 2019.

[CIT0046] Maxwell FG , Jennings PR. Breeding plants resistant to insects. Environmental science and technology. USA: John Wiley & Sons Inc; 1980.

[CIT0047] Murúa G , Molina-Ochoa J, Coviella C. Population dynamics of the fall armyworm, *Spodoptera frugiperda* (Lepidoptera: Noctuidae) and its parasitoids in northwestern Argentina. Fla Entomol. 2006:89(2):175–182. 10.1653/0015-4040(2006)89[175:pdotfa]2.0.co;2

[CIT0048] Nboyine JA , Kusi F, Abudulai M, Badii BK, Zakaria M, Adu GB, Haruna A, Seidu A, Osei V, Alhassan S, et al. A new pest, *Spodoptera frugiperda* (J.E. Smith), in tropical Africa: its seasonal dynamics and damage in maize fields in northern Ghana. Crop Prot. 2020:127(January 2020):104960. 10.1016/j.cropro.2019.104960

[CIT0049] Niassy S , Agbodzavu MK, Kimathi E, Mutune B, Abdel-Rahman EFM, Salifu D, Hailu G, Belayneh YT, Felege E, Tonnang HEZ, et al. Bioecology of fall armyworm *Spodoptera frugiperda* (J. E. Smith), its management and potential patterns of seasonal spread in Africa. PLoS One. 2021:16(6):e0249042. 10.1371/journal.pone.0249042 34115755 PMC8195398

[CIT0050] ONACC. Pluviometrie et temperature dans la regions sud ouest du Cameroun. In: Analyse de l’evolution de 1950 à 2015 et projection jusqu’ à 2090; Yaoundé, Cameroun; 2028.

[CIT0051] Osae MY , Frimpong JO, Sintim JO, Offei BK, Marri D, Ofori SEK. Evaluation of different rates of Ampligo insecticide against fall armyworm (*Spodoptera frugiperda* (JE Smith); Lepidoptera: Noctuidae) in the coastal savannah Agroecological zone of Ghana. Adv Agric. 2022:2022(1):1–14. 10.1155/2022/5059865

[CIT0052] Otim HM , Fiaboe KKM, Akello J, Mudde B, Obonyom AT, Bruce AY, Opio WA, Chinwada P, Hailu G, Paparu P. Managing a transboundary pest: the fall armyworm on maize in Africa. In: Moths and caterpillars. IntechOpen; 2021 [accessed 2024 May13]. 10.5772/intechopen.96637

[CIT0053] Overton K , Maino JL, Day R, Umina PA, Bett B, Carnovale D, Ekesi S, Meagher R, Reynolds OL. Global crop impacts, yield losses and action thresholds for fall armyworm (*Spodoptera frugiperda*): a review. Crop Prot. 2021:145(July 2021):105641. 10.1016/j.cropro.2021.105641

[CIT0054] Phillippe R , de Berchoux C, Mariau D. Les techniques de traitements dans les plantations de palmiers à huile en Côte d’Ivoire: Méthodes et appareillages. Oléagineux. 1983:38(6):349–363.

[CIT0055] Ponnusamy K. Farmers’ participatory assessment of neem-based insecticide in controlling the ear head bug (*Leptocorisa acuta*) in rice. Madras Agric J. 2003:90(7-9):564–566.

[CIT0056] Prasanna B , Huesing JE, Eddy R, Peschke VM. Fall armyworm in Africa: a guide for integrated pest management. Mexico: USAID; CIMMYT; 2018.

[CIT0058] Prasanna B , Huesing JE, Peschke VM, Eddy, editors. Fall armyworm in Asia: a guide for integrated pest management. Mexico (CDMX): CIMMYT; 2012.

[CIT0057] R Core Team. R: a language and environment for statistical computing. Vienna, Austria: R Foundation for Statistical Computing; 2023. https://www.Rproject.org/.

[CIT0059] Rigby RA , Stasinopoulos DM. Generalized additive models for location, scale, and shape. J R Stat Soc Ser C Appl Stat. 2005:54(3):507–554. 10.1111/j.1467-9876.2005.00510.x

[CIT0060] Shiferaw B , Prasanna BM, Hellin J, Bänziger M. Crops that feed the world 6. Past successes and future challenges to the role played by maize in global food security. Food Secur. 2011:3(3):307–327. 10.1007/s12571-011-0140-5

[CIT0061] Silvain JF , Ti-A-Hing J. Prediction of larval infestation in pasture grasses by *Spodoptera frugiperda* (Lepidoptera: Noctuidae) from estimates of adult abundance. Fla Entomol. 1985:68(4):686. 10.2307/3494875

[CIT0062] Sims SR. Influence of Soil type and rainfall on pupal survival and adult emergence of the fall armyworm (Lepidoptera: Noctuidae) in Southern Florida. J Entomol Sci. 2008:43(4):373–380. 10.18474/0749-8004-43.4.373

[CIT0063] Soha ME-D. The partial budget analysis for sorghum farm in Sinai Peninsula, Egypt. Ann Agric Sci. 2014:59(1):77–81. 10.1016/j.aoas.2014.06.011

[CIT0064] Sokame BM , Musyoka B, Obonyo J, Rebaudo F, Abdel-Rahman EM, Subramanian S, Kilalo DC, Juma G, Calatayud P-A. Impact of an exotic invasive pest, *Spodoptera frugiperda* (Lepidoptera: Noctuidae), on resident communities of pest and natural enemies in maize fields in Kenya. Agronomy. 2021:11(6):1074. 10.3390/agronomy11061074

[CIT0065] Tefera T , Mugo S, Mwimali M, Anani B, Tende R, Beyene Y, Gichuki S, Oikeh SO, Nang'ayo F, Okeno J, et al. Resistance of Bt-maize (MON810) against the stem borers *Busseola fusca* (Fuller) and *Chilo partellus* (Swinhoe) and its yield performance in Kenya. Crop Prot. 2016:89(November 2016):202–208. 10.1016/j.cropro.2016.07.023 27812235 PMC5026401

[CIT0066] Tshiabukole JPK , Khonde GP, Phongo AM, Ngoma N, Kankolongo AM, Vumilia RK, Djamba AM. Simulation of fall armyworm (*Spodoptera frugiperda*) attacks and the compensative response of quality protein maize (*Zea mays*, var. Mudishi-1 and Mudishi-3) in Southwestern DR Congo. OAlib. 2021:8(3):1–14. 10.4236/oalib.1107217

[CIT0067] van den Berg J , Britz C, du Plessis H. Maize yield response to chemical control of *Spodoptera frugiperda* at different plant growth stages in South Africa. Agriculture. 2021:11(9):826. 10.3390/agriculture11090826

[CIT0068] World Bank. Eastern Africa – a study of the regional maize market and marketing costs. Washington (DC, USA): World Bank Publications, World Bank; 2009. http://hdl.handle.net/10986/3155

